# Photoacidity and ESIPT in luteolin: acidity-vibrational frequency relationships across electronic states

**DOI:** 10.1039/d6ra05576a

**Published:** 2026-09-24

**Authors:** Siramol Photiganit, Ananda Thongyu, Pannipa Panajapo, Jittima Thisuwan, Kritsana Sagarik

**Affiliations:** a School of Chemistry, Institute of Science, Suranaree University of Technology Nakhon Ratchasima 30000 Thailand kritsana@sut.ac.th; b Division of Science, Faculty of Education, Nakhon Phanom University Nakhon Phanom 48000 Thailand thisuwan@gmail.com

## Abstract

The photochemical antioxidant properties of small organic molecules in electronically excited states have become an important topic in modern photochemistry and redox biology. Photoexcitation can trigger ultrafast proton and hydrogen transfer processes, leading to substantial changes in acidity and redox behavior. This study systematically explores the excited-state proton/hydrogen transfer pathways, acidity, and photophysical characteristics of luteolin (LU), a compound containing multiple potential deprotonation sites, using density functional theory (DFT) and time-dependent DFT (TD-DFT) calculations. Validation against previously reported theoretical and experimental data demonstrates that the employed DFT and TD-DFT/B3LYP/aug-cc-pVDZ methodology provides a reliable and consistent description of the molecular structure, energetics, and photophysical behavior of LU in both the ground (S_0_) and excited (S_1_) states. To evaluate acidity, the p*K*_a_ and 
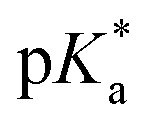
 values are determined using three distinct yet complementary computational models: gas phase (isolated LU molecule), an implicit solvation model, and an explicit solvation model (LU–H_2_O hydrogen-bonded complexes). The close agreement among these models confirms that they can be used in a complementary manner to reliably investigate systems with multiple deprotonation sites, offering a comprehensive picture of acid–base properties across different environments. The DFT and TD-DFT calculations reveal that the intramolecular hydrogen bond and the associated excited-state proton transfer (ESIPT) involving the benzopyran-ring O–H group (LU-C5) directly influence the vibrational properties and acidity of the most acidic catecholic O–H group (LU-C4′). Additionally, vibrational analysis reveals that near-linear correlations between O–H stretching frequencies and both p*K*_a_ and 
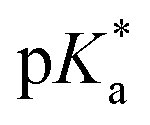
 persist in all three models. To the best of our knowledge, these results provide the first evidence that such vibrational descriptors remain valid across different solvation environments and electronic states, offering a practical approach for predicting photoacidity. Overall, this study provides detailed insights into the excited-state deprotonation behavior and photophysical properties of LU while establishing transferable computational strategies for investigating excited-state proton transfer in complex photoactive systems.

## Abstract 2

Complementary model for p*K*_a_ calculations in the ground and excited electronic states.

## Graphical abstract

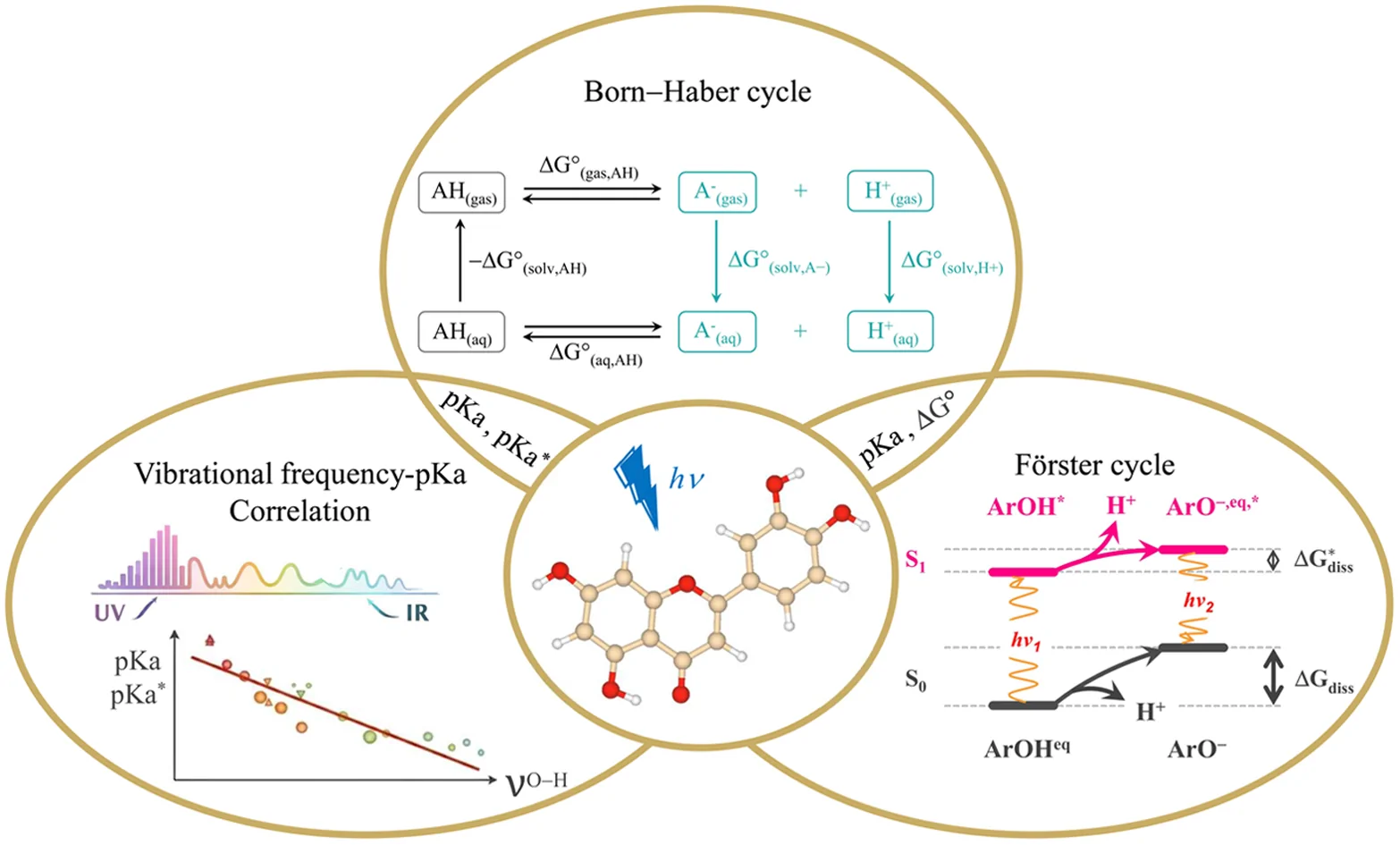

## Introduction

The antioxidant behavior of small photoactive molecules in their excited electronic states has emerged as a frontier in photochemistry and redox biology.^1^ Upon photoexcitation, molecules such as phenols, coumarins, and naphthols undergo dramatic shifts in the logarithm of the acidity constant 
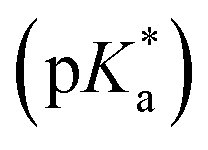
 and redox potential, enabling ultrafast hydrogen-atom transfer (HAT), electron transfer (ET), or proton-coupled electron transfer (PCET) to reactive oxygen species such as peroxyl radicals (ROO˙).^2–4^ These processes are central to understanding the mechanism by which light-activated antioxidants mitigate oxidative stress in biological or material systems.

In the ground electronic state, conventional phenolic antioxidants mainly quench radicals through HAT governed by the O–H bond dissociation energy (BDE).^2,4^ Photoexcitation reduces the effective O–H BDE and increases the driving force for PCET.^5^ The excited-state acidity, quantified by 
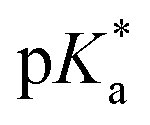
, typically decreases by 6–10 units relative to the ground state acidity, converting weak acids (*e.g.*, phenols, p*K*_a_ ≈ 10) into strong photoacids 
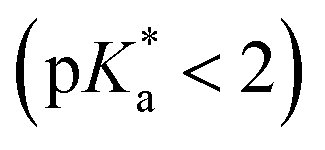
. This shift promotes deprotonation and generates an excited phenoxide species (ArO^−,^*), which serves as a more potent electron donor to ROO^˙^ radicals.^3^

In general, excited-state proton transfer (ESPT) occurs on femtosecond to picosecond timescales, with solvent-assisted pathways dominating in aqueous and protic environments.^6^ Super-photoacids, such as 5,8-dicyano-2-naphthol, exhibit 
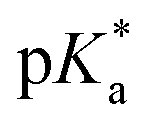
 values below −4, demonstrating nearly barrierless ESPT and providing model systems for probing the limits of excited-state deprotonation.^3^ Studies have revealed that solvent polarity and hydrogen bonds (H-bonds) strongly influence both ESPT efficiency and subsequent electron-transfer or HAT reactivity toward radicals. Theoretical approaches based on density functional theory (DFT) and time-dependent DFT (TD-DFT) have been instrumental in quantifying a range of excited-state properties and processes, including ESPT reactions.^7–11^

Flavonoids have been extensively employed in the design and synthesis of photosensitizers for dye-sensitized solar cells. More than 5000 types of flavonoids have been isolated from plants.^12^ In nature, flavonoids generally occur in the form of glycosides. However, theoretical and experimental investigations of the relationships between structural and vibrational properties and the acid–base behavior of flavonoids, particularly in electronically excited states, remain relatively limited.^13,14^

Luteolin (5,7,3′,4′-tetrahydroxyflavone, abbreviated as LU herein), a compound belonging to the flavonoid group, has been reported to reduce brain degeneration and impede memory decline associated with aging. LU is found in various plants such as carrot, peppers, celery, olive oil, peppermint, rosemary, and chamomile.^15–17^ Previous studies have demonstrated that the anticancer properties of LU are associated with apoptosis in cancer cells. Furthermore, LU has the ability to cross the blood–brain barrier, making it useful in treating central nervous system disorders, including brain cancers.^18^ More recently, LU-containing formulations have been proposed as potential therapeutic agents for alleviating brain fog associated with long-COVID syndrome and cognitive dysfunction associated with chemotherapy (chemofog).^19^ LU, which possesses a structural framework essential for antioxidant activity, plays a crucial role in biological and biochemical reactions. Its antioxidant activity primarily arises from the hydroxyl (O–H) groups at the 3′ and 4′ positions of the catechol ring, the C_2_=C_3_ double bond, and the carbonyl (C=O) group at the C_4_ position of the benzopyran ring (Fig. 1).^20^

**Fig. 1:**
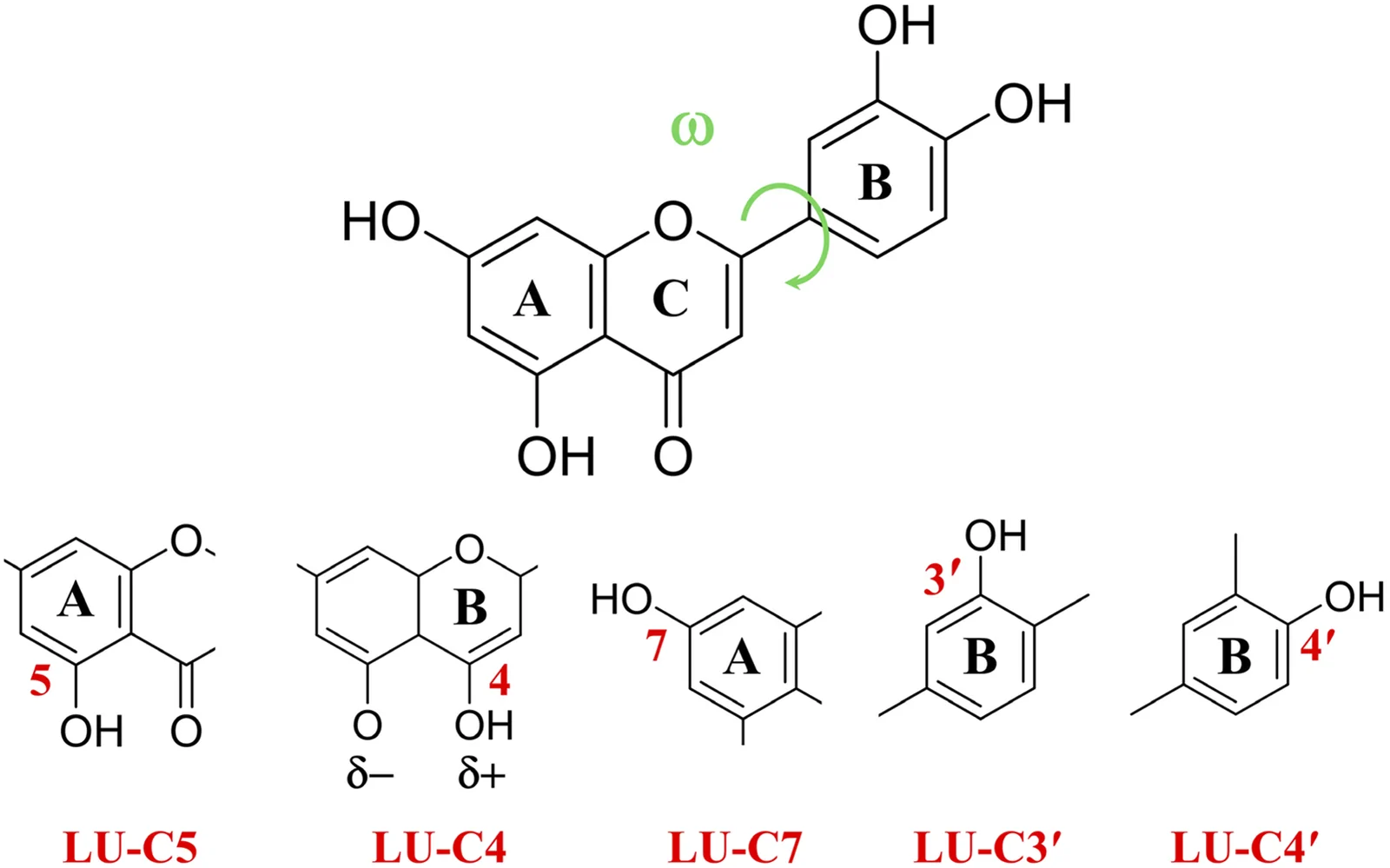
Structures and numbering system of the four deprotonation sites of luteolin (LU). AC = benzopyran ring; B = catechol ring. *ω* = dihedral angle between benzopyran and catechol rings.

Given the crucial roles played by acid–base reactions and proton transfer/HAT processes in the chemical and biochemical behavior of LU, several studies have investigated the relationships among its p*K*_a_, pH, and physicochemical properties. Experimental results have indicated that in the ground electronic state, the proton dissociation behavior strongly depends on the position of the O–H groups on the benzopyran and catechol rings.^21,22^ However, for the excited electronic state, no study has clearly reported the sequential order of proton dissociation at the various hydroxyl sites that directly correlates with proton transfer or HAT reactions.^23^

For the ground electronic state, Herrero-Martínez *et al.*^23^ examined the dissociation order and acidity constants of flavonols using capillary electrophoresis. Their experimental results revealed that for flavanols such as catechin and epicatechin, whose molecular structures are similar to that of LU, the first ionization occurs on the phenyl C ring, whereas the second ionization occurs on the phenyl A ring. By contrast, for flavonols such as morin, the first proton dissociation occurs on the phenyl A ring, followed by the second dissociation on the phenyl B ring. These observations indicate that the proton dissociation order cannot be reliably inferred from structural similarity alone and should instead be established on a case-by-case basis.

Amat *et al.*^14^ investigated the acid–base behavior of LU by analyzing the potential energy surface (PES) in an aqueous dielectric environment using the DFT/6-311++G** method. Their calculations indicated that proton dissociation occurs most readily at the 4′ position (LU-C4′) on the catechol ring. This preference arises from the torsional rotation between the benzopyran (AC) and catechol (B) rings (*ω* in Fig. 1), which alters molecular geometry and electron density distribution. Consequently, the O–H group at LU-C4′ plays a more important role in proton transfer reactions than other hydroxyl sites. However, the p*K*_a_ calculated using the DFT/6-311++G** approach showed only limited agreement with experimental values. This discrepancy arose because the model considered only a continuum dielectric environment,^14^ without explicitly accounting for the probability of H-bond formation involving O–H groups. Such direct H-bond interactions are particularly important in aqueous systems and can significantly influence both proton dissociation and the stabilization of deprotonated species.

In another study, the acid–base and spectroscopic properties of fisetin and LU were studied using theoretical and experimental methods.^24^ The fluorescence emission of these flavonoids was ascribed to an excited-state intramolecular proton transfer (ESIPT) process, which alters molecular configuration and lowers emission energy. Because of structural differences, only one proton-transfer pathway is possible in each molecule: H3 transfer in fisetin and H5 transfer in LU. The emission wavelengths calculated at the TD-DFT/M06-2X/6-31++G(d,p) level agreed with fluorescence spectra measured in both powders and solutions, supporting the occurrence of the ESIPT mechanism.

In our previous study,^7^ complementary theoretical approaches were applied to elucidate the photodecarbonylation mechanism of flavonol-1 (3-hydroxy-2-phenyl-4*H*-benzo[*g*]chromen-4-one), which is an organic, photoactive, carbon monoxide-releasing molecule with potential as a dual-function photosensitizer. Combined DFT and TD-DFT analyses revealed, for the first time, that double ESIPT processes occurring in the S_1_ state played a central role in driving key acid–base reactions. The results further showed that solvent polarity strongly modulated the competition between fluorescence relaxation and intersystem crossing (ISC). Moreover, kinetic and thermodynamic evaluations indicated that the aerobic reaction pathways leading to salicylic acid ester formation and CO release were essentially barrierless and energetically favorable, which accounted for their higher chemical yields relative to the anaerobic pathway that produced lactone and CO. Importantly, intersection geometries at the S_1_/T_1_, S_1_/S_0_, and T_1_/S_0_ crossings were identified as reactive precursors that promote rapid ISC, thereby facilitating both singlet-oxygen (^1^O_2_) sensitization and CO photo release.

Based on the above discussion, although several aspects of the acidity, ESIPT, and photophysical properties of LU have been investigated, a systematic site-specific description connecting ground- and excited-state acidities with vibrational properties, ESIPT, and solvent effects remains limited in both theoretical and experimental studies. To address this gap, the present work investigates the photoacidity, ESIPT mechanisms, and photophysical properties of LU in both the gas phase and aqueous solution using theoretical approaches. The applied computational methods are first assessed by benchmarking the calculated photophysical properties against available experimental data.

Quantum chemical calculations, reaction pathway optimizations, and three complementary approaches—the Born–Haber cycle, vibrational frequency – p*K*_a_ correlations, and the Förster cycle—are employed to determine the ground- and excited-state acidity constants (p*K*_a_ and 
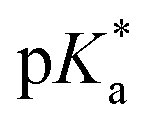
, respectively) for multiple deprotonation sites of LU, considering both continuum solvation and explicit solvent effects *via* 1 : 1 LU–H_2_O complexes. Particular emphasis is placed on assessing the consistency of these computational approaches for predicting p*K*_a_ and 
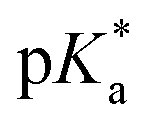
, while elucidating the influence of ESIPT on excited-state acidity across solvents spanning a wide polarity range. The insights gained may guide the synthesis, design, and enhancement of LU-based compounds with improved properties and will contribute valuable knowledge to the fields of pharmacology, medicine, nutraceuticals, and drug development.

## Computational methods

### Quantum chemical calculations

Because numerous notations and symbols are used throughout the manuscript, they are categorized and summarized in Table S1 to keep the discussion concise and facilitate readability. DFT and TD-DFT calculations were performed using the ORCA software package^25,26^ with the Tamm–Dancoff approximation applied within the TD-DFT framework.^27^ The B3LYP hybrid functional^28,29^ combined with the aug-cc-pVDZ basis set^30^ was employed for both DFT and TD-DFT computations, following previous benchmark studies on photochemical reactions,^8,10^ including systems such as boron dipyrromethene^31^ and BF_2_-formazanate-based photosensitizers.^11^ Benchmark comparisons with the CASPT2 method for glycine photodissociation and formation^8,10^ indicated that DFT/B3LYP and TD-DFT/B3LYP yielded structures and energies on the S_0_ and S_1_ PESs consistent with CASPT2 results. Solvent effects were modeled using the conductor-like polarizable continuum model (CPCM),^32,33^ with dielectric constants (*ε*) of 1 and 80 representing the gas phase and aqueous solution, respectively.

In the present work, because the experimental p*K*_a_ measurements were performed in mixed-solvent systems (*e.g.*, methanol/water in ref. 14), for which the effective local dielectric constant is not well defined, the gas phase (*ε* = 1) and aqueous continuum (*ε* = 80) were considered as limiting dielectric environments to assess the effects of solvent polarity on the calculated p*K*_a_ and 
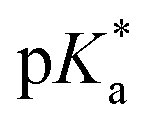
 values. Intermediate dielectric constants, such as *ε* ≈ 33 for pure methanol, were therefore not explicitly considered. Solvent effects were treated using the linear-response conductor-like polarizable continuum model (LR-CPCM) implemented in ORCA, in combination with TD-DFT for the excited-state calculations.

All LU conformers that could be involved in the ground (S_0_) and excited-state (S_1_ and T_1_) ESIPTs were optimized using the DFT and TD-DFT/B3LYP/aug-cc-pVDZ methods with *ε* = 1 and 80. The equilibrium structures, highest occupied molecular orbital (HOMO)-lowest unoccupied molecular orbital (LUMO) distributions, and total and excitation energies are presented in Tables S2 and S3. Natural transition orbital (NTO) analyses were also performed at selected structures along the excited-state PES using TD-DFT/B3LYP/aug-cc-pVDZ to characterize the electronic nature of the relevant excited states.

### Photophysical property calculations

The UV-vis absorption spectra were simulated using the Excited State Dynamics [ESD(ABS)] protocol implemented in ORCA, rather than by simply applying Gaussian or Lorentzian broadening to the calculated vertical electronic transitions. The excited-state potential-energy surfaces were treated within the Vertical Gradient (VG) approximation, using the ground-state Hessian and the excited-state gradient evaluated at the ground-state equilibrium geometry.^34^ Vibronic effects were included through both Franck–Condon and Herzberg–Teller contributions.

All fluorescence, phosphorescence, and ISC calculations were performed using the corresponding ESD protocols implemented in ORCA. The fluorescence spectra and radiative rate constants (*k*^fluor^) were calculated using the ESD(FLUOR) protocol. The calculations employed the optimized S_1_ geometry together with the ground- and excited-state Hessians to account explicitly for vibrational structure. Both Franck–Condon and Herzberg–Teller contributions were included, and the spectral profiles were generated using a Voigt lineshape with Lorentzian and Gaussian broadening parameters of 75 and 200 cm^−1^, respectively. For CPCM calculations, the fluorescence rate was evaluated with the refractive-index correction implemented in the ESD formalism.^34^

The phosphorescence rate constants (*k*^phos^) were calculated using the ESD(PHOSP) protocol. Spin–orbit coupling (SOC) was treated using the resolution-of-identity spin–orbit mean-field (RI-SOMF) approach within full TD-DFT, and the ground- and triplet-state Hessians were included to account for vibronic effects. Herzberg–Teller contributions were explicitly included, and the corresponding T_1_ → S_0_ energy separation was supplied through the ESD energy–gap parameter.^35^ The S_1_ → T_1_ intersystem-crossing rate constants (*k*^ISC^) were calculated using the ESD(ISC) protocol within a Fermi-golden-rule/path-integral formalism. Spin–orbit coupling, the S_1_ and T_1_ Hessians, Duschinsky rotation, and Herzberg–Teller vibronic coupling were explicitly included, and the S_1_ → T_1_ adiabatic energy difference was supplied from the corresponding optimized-state energies.^35^

The S_1_ → S_0_ energy gap (Δ*E*^S_1_→S_0_^) was evaluated at the optimized S_1_ equilibrium geometry without explicit optimization of an S_1_/S_0_ crossing structure. The S_1_/T_1_ region was further investigated by geometry optimization of the SOC state at the TD-DFT/B3LYP/aug-cc-pVDZ level using the ORCA IROOT 1 and SOCGRAD TRUE keywords. The resulting SOC-optimized structure was used for subsequent evaluation of the S_1_/T_1_ energy separation and ISC process.

### ESIPT/HAT pathways

Because the S_0_ → T_1_ transition is spin-forbidden, excited-state PES calculations were performed for the reactions occurring in the S_1_ state. Accordingly, the S_1_ PESs were initially calculated starting from the equilibrium structure in the S_0_ state using the TD-DFT/B3LYP/aug-cc-pVDZ and nudged elastic band (NEB) methods.^36^ With *ω* varying from 0° to 180°, the vertical excitation energy (Δ*E*^S_0_→S_1_^), energy barriers (Δ*E*^‡^), and key stationary points—including the equilibrium and transition structures, as well as the structures in the S_1_/S_0_ and S_1_/T_1_ crossing regions—were identified and refined.

Around seven to eight representative geometries on the S_1_ PES were optimized at the TD-DFT/B3LYP/aug-cc-pVDZ level using the ORCA software package. To confirm the applicability of the DFT and TD-DFT/B3LYP/aug-cc-pVDZ methods, important photophysical properties, such as fluorescence, phosphorescence, and ISC rates, were computed and benchmarked against reported theoretical and experimental data. The theoretical strategies and methods employed to study ESIPTs are presented in Fig. 2a.

**Fig. 2:**
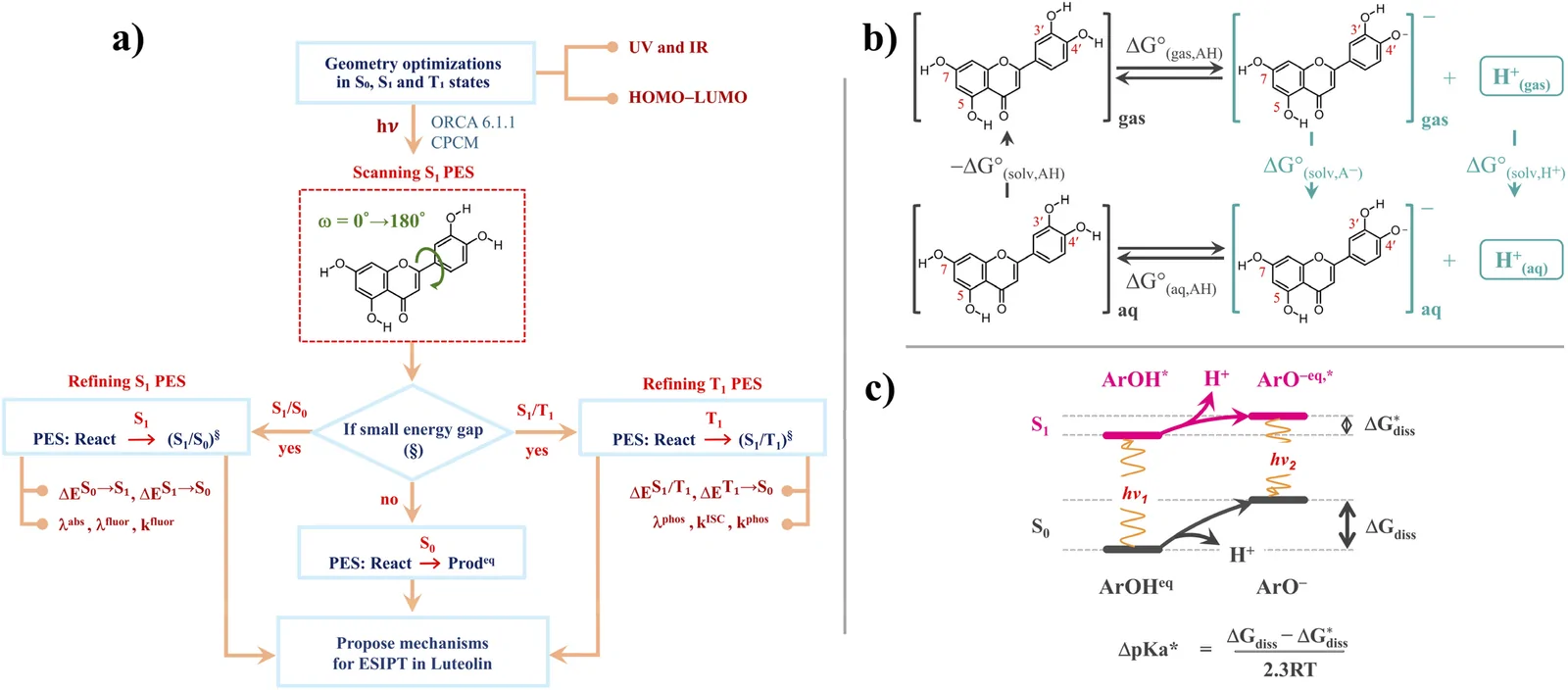
(a) Theoretical methods used in the study on the photoacidity and ESIPT of LU. (b) Example of the Born–Haber cycle model used in the calculations of p*K*_a_ of LU (LU-C4′) in the S_0_ state using the DFT/B3LYP/aug-cc-pVDZ method. (c) The Förster cycle. 
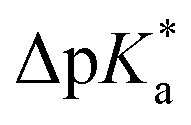
 = change in acidity upon S_0_ → S_1_ excitation; ArOH^eq^ = equilibrium structure of aromatic hydroxyl compound in the S_0_ state; ArO^−eq,^* = equilibrium structure of deprotonated aromatic hydroxyl compound in the S_1_ state. Δ*G*_diss_ and 
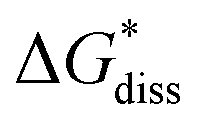
 = Gibbs free energy changes for the deprotonation in the ground (S_0_) and excited electronic (S_1_) states, respectively. Notations and symbols are defined in Table S1.

### Calculations of p*K*_a_ and 
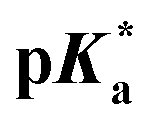


To capture the effects of local H-bonding in the first solvation shell while avoiding the increased complexity associated with larger water clusters, a single explicit water molecule was introduced at each deprotonation site. Accordingly, three computational models were rigorously tested against available experimental data: (i) an isolated-LU model (gas phase with *ε* = 1); (ii) an implicit solvation model of LU (aqueous solution with *ε* = 80); and (iii) an explicit solvation model (1 : 1 LU–H_2_O complexes), characterized by LU forming an intermolecular O–H⋯O H-bond with a single water molecule at the LU-C5, LU-C7, LU-C3′, and LU-C4′ positions.

It should be noted that a combined explicit–implicit (cluster-continuum) model may provide a more complete description of solvation. In the present work, however, the two solvent models were intentionally treated separately to distinguish bulk dielectric effects from site-specific H-bonding effects. Combining the two treatments would introduce additional variables associated with the number, positions, and configurations of explicit water molecules, making their individual effects on the O–H stretching frequencies and acidity more difficult to distinguish.

#### Born–Haber cycle model

In p*K*_a_ calculations, the Born–Haber cycle relates the gas-phase deprotonation free energy and solvation free energies of the acid, base, and proton to compute the overall aqueous deprotonation Gibbs free energy (Δ*G*°), which is then converted into p*K*_a_*via* Δ*G*° = 2.303RT × p*K*_a_.^37,38^ In this work, both p*K*_a_ and 
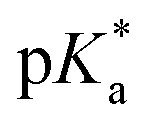
 were calculated using the Born–Haber cycle, for which the deprotonation at LU-C4′ in the S_0_ state is shown as an example in Fig. 2b. All the acid and conjugate-base structures involved in the Born–Haber cycle were primarily optimized using the DFT and TD-DFT/B3LYP/aug-cc-pVDZ methods with *ε* = 1 and 80 (Tables S2 and S3), following which the p*K*_a_ and 
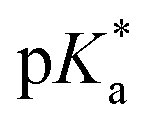
 values in the S_0_ and S_1_ states were computed using the Gibbs free energies in eqn (1)–(6).^38^1
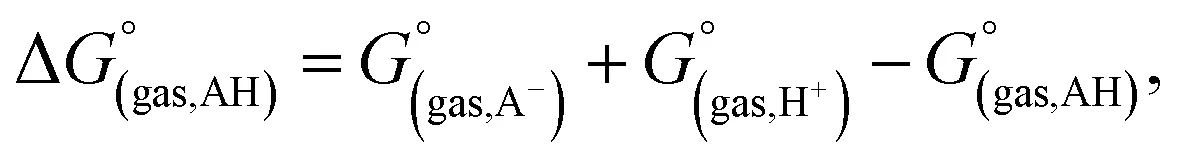
2
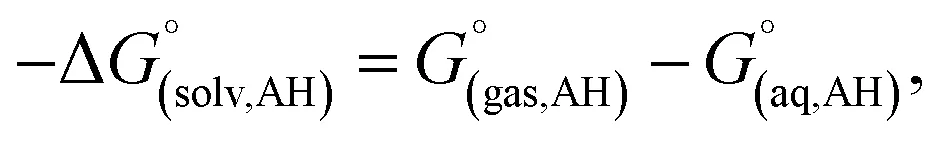
3
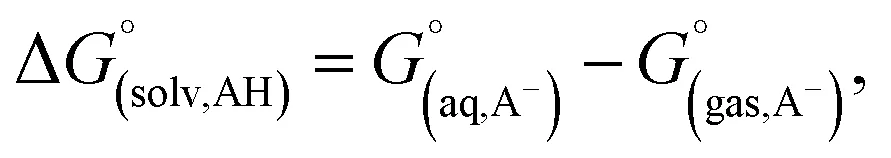
4
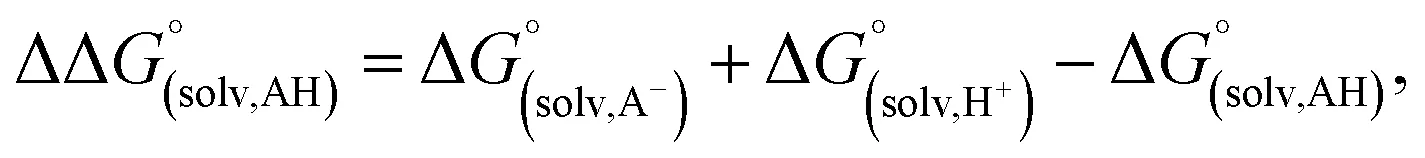
5
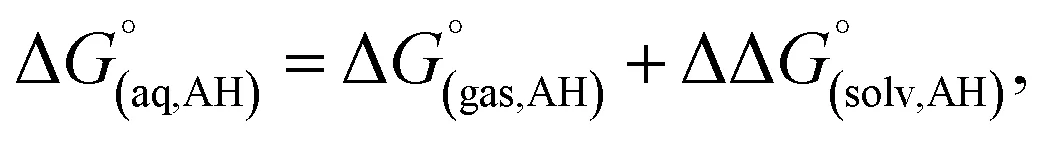
6
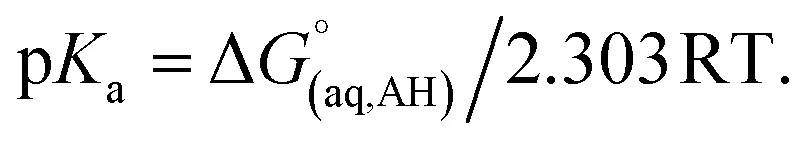


Herein, the experimental Gibbs free energies of the gas-phase and solvated protons, 
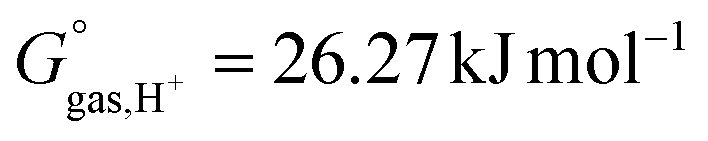
 and 
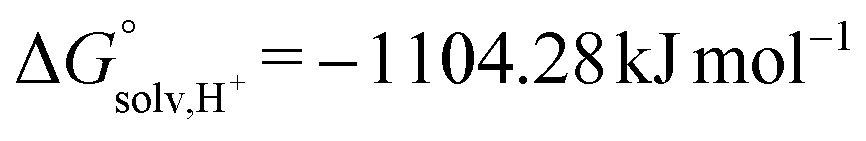
, respectively, were initially adopted.^39,40^ However, these quantities have been recognized as a major source of uncertainty in theoretical p*K*_a_ calculations, and previous studies have highlighted deficiencies associated with their direct use in conjunction with specific electronic-structure methods.^38^ Therefore, to improve the reliability and internal consistency of the predicted p*K*_a_ values, we sought to identify proton free-energy values that are more appropriate for the theoretical approaches employed in the present work.

#### Vibrational frequency – p*K*_a_ correlation model

Previous theoretical and experimental studies demonstrated the feasibility of applying vibrational frequency – p*K*_a_ correlations in the ground electronic state.^41,42^ However, information regarding their applicability in the excited electronic states remains limited. For the ground electronic state, empirical and theoretical studies have demonstrated that local vibrational frequencies—especially those associated with bond stretches directly involved in proton transfer—strongly correlate with p*K*_a_ values.^41^

Herein, vibrational frequency – p*K*_a_ correlations were systematically investigated by computing the O–H stretching frequencies (*ν*^O–H^) for all the potential deprotonation sites at *ε* = 80 and *ε* = 1. The calculations were performed using the ORCA software package. The resulting *ν*^O–H^ were then correlated with p*K*_a_ values derived from the Born–Haber cycle. The same computational strategies were applied to estimate the vibrational frequency – 
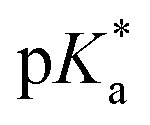
 correlation using the O–H stretching frequencies in the S_1_ state.

#### Förster cycle model

For common photoacids, computational thermodynamic cycles based on Förster's relation^43^ and continuum solvent models can reproduce experimental 
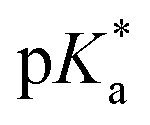
 values within 1–2 units.^44^ Therefore, attempts were made to apply a Förster cycle model (Fig. 2c) to calculate the 
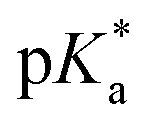
 of LU using eqn (7) and (8);^3^ theoretical studies based on DFT and TD-DFT methods have proven to be powerful tools for quantifying 
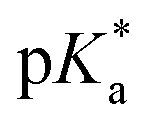
.^6^ One has7
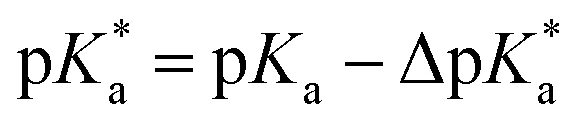
8
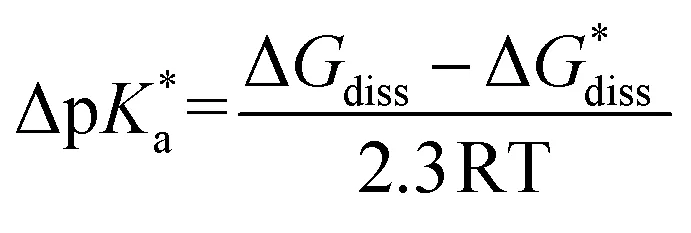


The p*K*_a_ in eqn (7) was obtained from the Born–Haber cycle model, whereas 
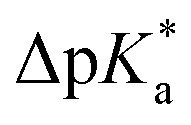
 represents the change in acidity upon S_0_ → S_1_ excitation. In the present Förster-cycle calculations, 
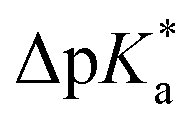
 was evaluated at *ε* = 80 using two equilibrium structures and the corresponding vertical photophysical energy terms: the vertical absorption energy (S_0_ → S_1_) evaluated at the S_0_ equilibrium structure of the neutral form and the vertical emission energy (S_1_ → S_0_) evaluated at the S_1_ equilibrium structure of the corresponding deprotonated form (Fig. 2c). Notably, Δ*G*_diss_ and 
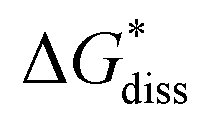
 represent the Gibbs free energy differences for proton dissociation at the LU-C5, LU-C7, LU-C3′, and LU-C4′ positions in the S_0_ and S_1_ states, respectively. These Gibbs free energies were directly obtained from vibrational frequency (Hessian) calculations using the ORCA software package.

## Results and discussion

Notations were introduced to discuss the equilibrium and key structures along the PESs. The superscripts (eq) and (*) indicate equilibrium and excited-state structures, respectively, while (‡) and (§) denote transition structures and those located at S_1_/T_1_ intersections. The superscript (*ε*) denotes the structures at *ε* = 80. Characteristic structures on the S_0_ and S_1_ PESs are labeled as Gr-[*n*]^eq^ and E-[*n*]^eq,^*, respectively, where Gr and E denote the ground- and excited-state structures, respectively. Hereafter, [*n*] denotes different conformational structures of a given LU species, distinguished primarily by the catechol-ring dihedral angle, *ω*. Specifically, Gr-[1]^eq^ and E-[1]^eq,^* denote the equilibrium structures at *ε* = 1 in the S_0_ and S_1_ states, respectively. To enable direct comparison with experimental data, thermodynamic energies were reported in kJ mol^−1^, while photophysical energies were expressed in eV and nm.

### Equilibrium structures

The equilibrium structures of LU and its site-specific deprotonated forms, obtained using the DFT and TD-DFT/B3LYP/aug-cc-pVDZ methods, are presented in Tables S2 and S3, respectively. Because the equilibrium structures of LU were not significantly different—except the O5–H5 → O4 ESIPT occurring in the intramolecular H-bond (LU-C5) for the molecule in the S_1_ state—the structures in the S_0_ state at *ε* = 80 are discussed first. In the next subsection, the equilibrium structures in the S_1_ and T_1_ states are analyzed in relation to the photophysical properties along the relaxation pathways. The DFT/B3LYP/aug-cc-pVDZ results (Fig. 3) revealed three similar equilibrium structures at *ε* = 80 and 1, namely, two planar structures (Gr-[1]^eq,*ε*^ and Gr-[3]^eq,*ε*^ with *ω* ≈ 0° and 180°, respectively) and one perpendicular structure (Gr-[2]^eq,*ε*^, *ω* ≈ 90°). These structures followed the same stability order in both environments; at *ε* = 80, the stability order was Gr-[1]^eq,*ε*^ ≈ Gr-[3]^eq,*ε*^ > Gr-[2]^eq,*ε*^, with the relative energy (Δ*E*^Rel^) values of 0.0, 0.06, and 18.2 kJ mol^−1^, respectively.

**Fig. 3:**
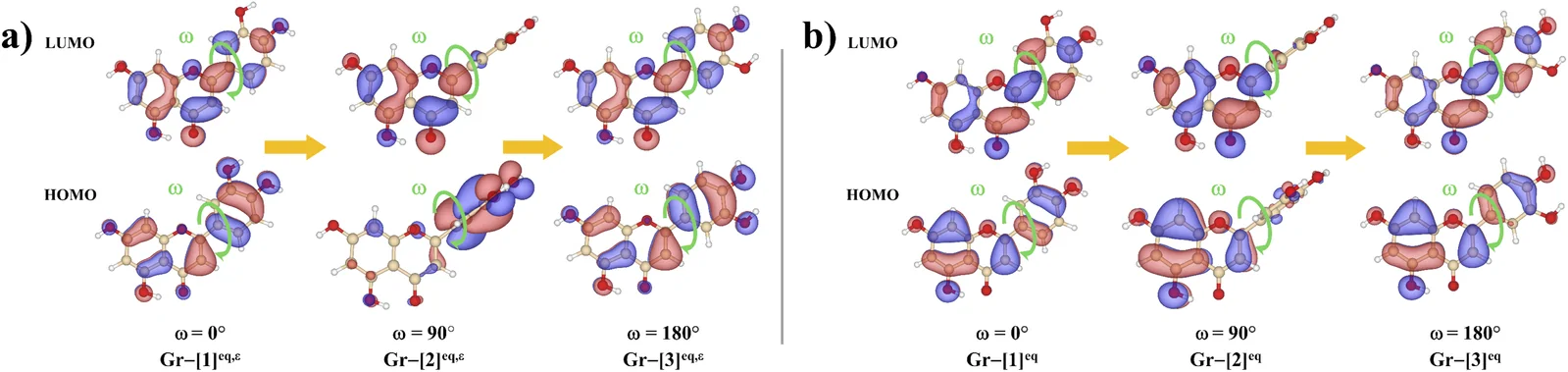
(a) and (b) Molecular orbitals (HOMO and LUMO distributions) of the equilibrium structures of luteolin in the S_0_ state in high and low local-dielectric environments (*ε* = 80 and 1, respectively), calculated using the DFT/B3LYP/aug-cc-pVDZ method. *ω* = dihedral angle between benzopyran and catechol rings. Notations and symbols are defined in Table S1.

Remarks should be made regarding the potential formation of intramolecular H-bonds in LU. Possible configurations involving O3′–H3′···O4′ and O4′–H4′···O3′ intramolecular H-bonds were examined using the DFT/M06-2X/6-31++G(d,p) method, with particular emphasis on conformational stability.^24^ Results indicated that in the S_0_ state, these conformers exhibited nearly identical stability, with differences of only ∼1 kJ mol^−1^ in total energy. Because this study focused on the lowest p*K*_a_ deprotonation site (LU-C4′),^14^ where the O4′–H4′ group remained available for H-bonding with water, the structure Gr-[1]^eq,*ε*^ was selected as the initial geometry for subsequent investigations.

### Analysis of the electronic character of the excited states

Analysis of the HOMO–LUMO distributions (Fig. 3a) showed that at *ε* = 80, while the electron density distribution in the LUMO remained similar to that at *ε* = 1, the HOMO exhibited a contrary trend: at *ω* = 90°, the electron density on the catechol ring increased, whereas that on the benzopyran ring decreased. By contrast, Fig. 3b reveals a general trend at *ε* = 1, where the rotation of the dihedral angle from *ω* = 0° to 90° led to a significant reduction in π-conjugation, accompanied by a corresponding redistribution of electron density in both the HOMO and LUMO. Specifically, for both orbitals, the electron density on the benzopyran ring increased, while that on the catechol ring decreased.

These findings suggested that the aromaticity of the catechol ring decreases upon S_0_ → S_1_ excitation and dihedral angle rotation in a polar solvent, implying that the photoexcitation-induced changes in the acidity of the O–H groups at LU-C3′ and LU-C4′ differed between *ε* = 80 and 1. Analysis of the photophysical properties (Table S2) indicated the same aromaticity trend, where the decrease in π-conjugation for the structure with *ω* = 90° led to an increase in S_0_ → S_1_ excitation energy.

For example, at *ε* = 80, the Δ*E*^S_0_→S_1_^ values were 3.55 and 3.76 eV for the structures Gr-[1]^eq,*ε*^ and Gr-[2]^eq,*ε*^, respectively, and 3.59 eV for the structure Gr-[3]^eq,*ε*^. The Δ*E*^S_0_→S_1_^ value of the structure Gr-[1]^eq,*ε*^ was in remarkable agreement with the π → π* transition observed in a spectrophotometric experiment in methanol (3.55 eV).^21^ The ultraviolet spectra (Fig. 4a and b) confirmed these excitation energies, with considerably higher oscillator strengths for the structures Gr-[1]^eq,*ε*^ and Gr-[2]^eq,*ε*^ (*i.e.*, at *ε* = 80) than for the structures Gr-[1]^eq^ and Gr-[2]^eq^ (*i.e.*, at *ε* = 1). Accordingly, the following discussion focuses on the results obtained at *ε* = 80.

**Fig. 4:**
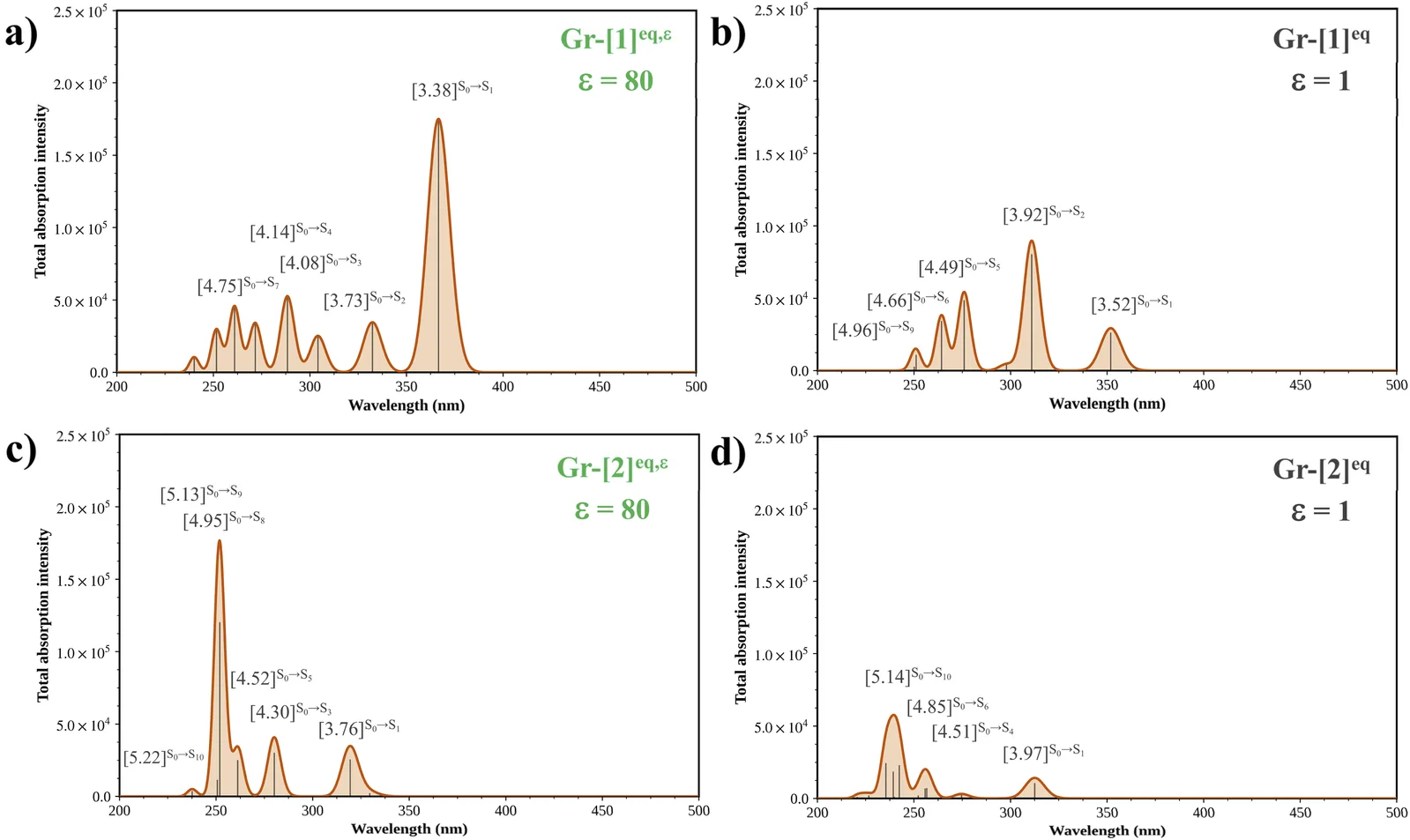
UV absorption spectra of the equilibrium structures of luteolin in the S_0_ state obtained from TD-DFT/B3LYP/aug-cc-pVDZ calculations. […]^S_0_→S_1_^ = vertical excitation in eV. (a) and (b) Structures Gr-[1]^eq,*ε*^ and Gr-[1]^eq^ (*ω* ≈ 0°) with *ε* = 80 and 1, respectively. (c) and (d) Structures Gr-[2]^eq,*ε*^ and Gr-[2]^eq^ (*ω* ≈ 90°) with *ε* = 80 and 1, respectively.

To further characterize the electronic state followed along the excited-state PES, NTO analyses were performed for representative structures at *ω* ≈ 0° and 90°, as well as for the *ω* ≈ 0° structure formed following O5–H5 → O4 ESIPT. In both the gas-phase and continuum-solvation models, the S_1_ excitation remains strongly dominated by the frontier-orbital HOMO → LUMO transition. The corresponding HOMO → LUMO contributions are 98.1 and 96.5% for Gr-[1]^eq^ and Gr-[2]^eq^, respectively, at *ε* = 1 (Table S4a), and 98.4 and 99.7% for Gr-[1]^eq,*ε*^ and Gr-[2]^eq,*ε*^, respectively, at *ε* = 80 (Table S4b). Notably, although ESIPT substantially changes the S_1_ excitation energy, the dominant frontier-orbital character remains essentially unchanged, with HOMO → LUMO contributions of 99.5 and 99.4% for E-[1]^eq,^* and E-[1]^eq,*ε*,^*, respectively, at *ε* = 1 and 80, respectively (Table S4c). These results indicate that both the *ω* rotation and ESIPT affect the energetics and structure of LU without substantially changing the dominant frontier-orbital character of the S_1_ state at the representative structures investigated along the excited-state PES.

To assess whether a single explicit water molecule provides an adequate representation of the local H-bonding effects, the S_0_ → S_1_ excitation energy was additionally calculated for a 1 : 4 LU–H_2_O complex, in which all four O–H groups of LU are H-bonded to water molecules. The resulting excitation energy in the gas phase of 3.48 eV (356 nm) (Fig. S1), compared with 3.57 eV (347 nm) for the 1 : 1 LU-C4′-H_2_O, indicates that the principal excitation energy is reasonably converged with respect to the number of explicitly included water molecules and supports the use of the 1 : 1 LU–H_2_O model for the present analysis.

### Relaxation pathways and photophysical properties

The potential energy profiles obtained from DFT and TD-DFT/B3LYP/aug-cc-pVDZ calculations, along with the NEB method, are shown in Fig. 5. The photophysical properties along the relaxation pathways are shown in Fig. 6. The potential energy profiles in the S_1_ state indicate that the rotational motion of the catechol ring within the Franck–Condon region (*ω* = 0°–1°) plays a significant role in the photophysical properties of LU. This motion modulates the excited-state PES, where radiative/nonradiative processes can occur. This behavior is consistent with that reported in our earlier study on flavonol-1 (a π-extended derivative of 3-hydroxyflavone), in which the rotation of the dihedral angle between the two aromatic rings within *ω* = 0°–1° led to double ESIPTs.^7^

**Fig. 5:**
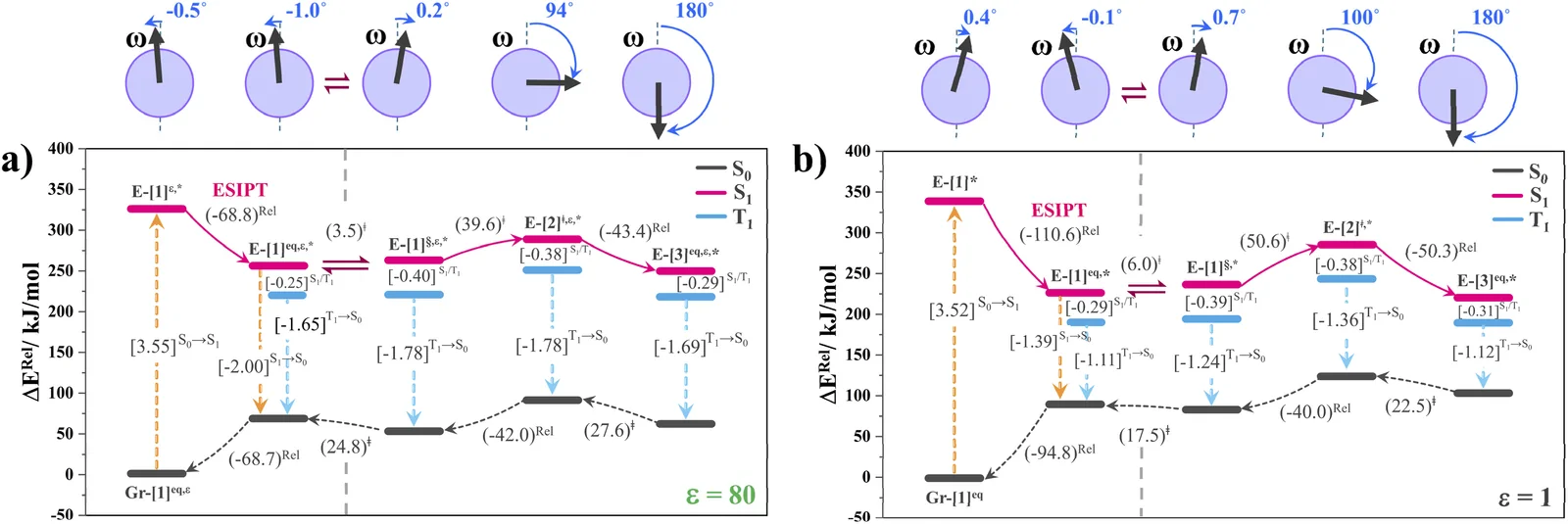
(a) and (b) The potential energy profiles with Newman projections for the vertical absorption (S_0_ → S_1_), fluorescence (S_1_ → S_0_), intersystem crossing (S_1_/T_1_ ISC) and phosphorescence (T_1_ → S_0_) of the photoexcited LU in *ε* = 80 and 1, respectively. The solid lines represent the potential energy profile obtained from the NEB method, whereas the dashed lines denote the ones calculated using the geometries on the S_1_ profile. Notations and symbols are defined in Table S1. Δ*E*^Rel^ = relative energy with respect to the precursor in the S_0_ state in kJ mol^−1^; (…)^Rel^ and (…)^‡^ = relaxation energy and energy barrier on the potential energy profile in kJ mol^−1^; […]^S_1_→S_0_^, […]^S_0_→S_1_^ and […]^T_1_→S_0_^ = vertical excitation and relaxation energies in eV; […]^S_1_→T_1_^ = intersystem crossing in eV.

**Fig. 6:**
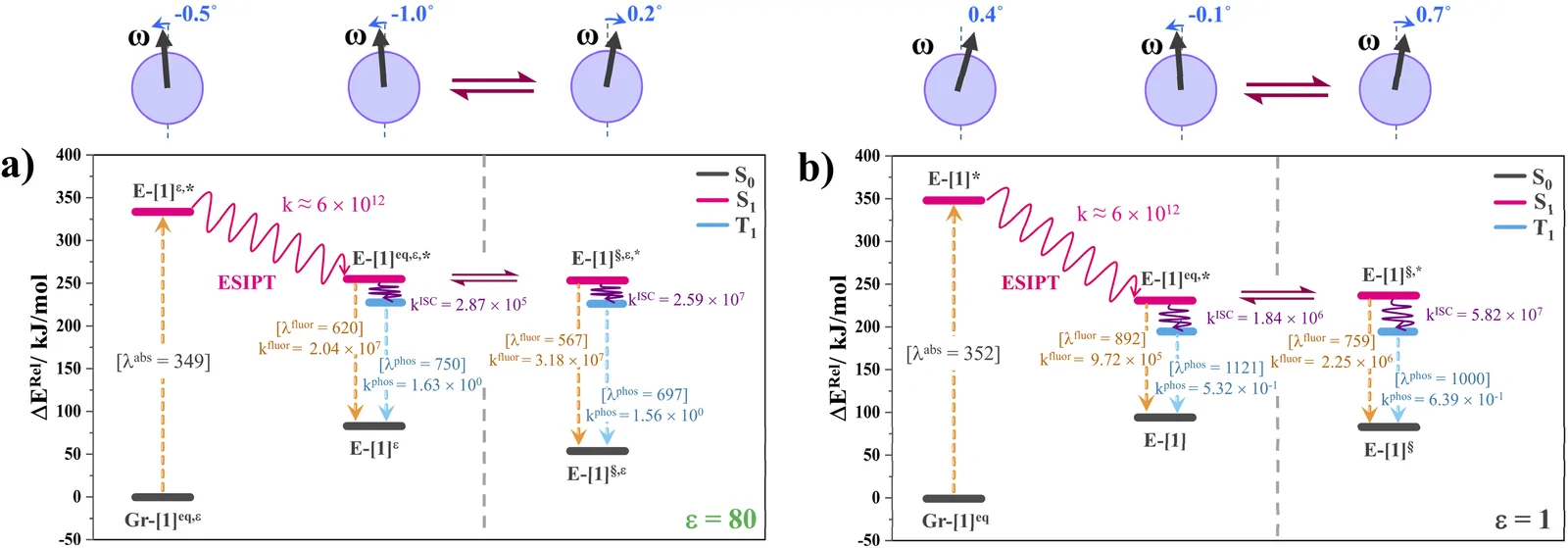
(a) and (b) Photophysical properties of LU on the potential energy profiles in Fig. 5 obtained from the TD-DFT/B3LYP/aug-cc-pVDZ method in *ε* = 80 and 1, respectively. Notations and symbols are defined in Table S1. Δ*E*^Rel^ = relative energy with respect to the precursor in the S_0_ state in kJ mol^−1^; [*λ* = …] = absorption or relaxation wavelength in nm; *k*^fluor^ and *k*^phos^ = fluorescence and phosphorescence rates in s^−1^, respectively; *k*^ISC^ = intersystem crossing rate in s^−1^.

For example, in polar solvent (*ε* = 80; Fig. 5a), the structure Gr-[1]^eq,*ε*^ was vertically excited to E-[1]^*ε*,^* with an excitation energy Δ*E*^S_0_→S_1_^ = 3.55 eV (*λ*^S_0_→S_1_^ = 349 nm). This S_0_ → S_1_ excited structure could subsequently relax to the equilibrium structure in the S_1_ state, structure E-[1]^eq,*ε*,^*, which was characterized by a planar structure with O5–H5 → O4 ESIPT along the intramolecular H-bond (LU-C5). The corresponding minimum-energy pathway along the O5–H5 → O4 proton-transfer coordinate reveals a barrierless S_1_ PES at *ε* = 80 (Fig. S2), with proton transfer from O5 to O4 leading to the proton-transferred structure denoted LU-C4 in Fig. 1. For this barrierless process, the characteristic rate estimated from the O–H stretching timescale is on the order of 10^12^–10^13^ s^−1^,^45^ consistent with an ultrafast ESIPT process.

From the structure E-[1]^eq,*ε*,^*, S_1_ → S_0_ radiative relaxation could occur, yielding a fluorescence energy Δ*E*^S_1_→S_0_^ = −2.00 eV (*λ*^fluor^ = 620 nm) with a fluorescence rate constant *k*^fluor^ = 2.04 × 10^7^ s^−1^ (Fig. 6a). The calculated fluorescence wavelength was in remarkable agreement with the reported values for LU powder and concentrated (2.6 mM) methanol solutions (*λ*^fluor^ ≈ 620 nm),^24^ confirming the proposed photo deactivation pathways. The calculated fluorescence rate constant was slightly lower than the corresponding value reported for flavonol-1,^7^ in which ultrafast double ESIPTs occurred in the S_1_ state, with *k*^fluor^ = 1.36 × 10^8^ s^−1^ at *ε* = 80.

Alternatively, the structure E-[1]^eq,*ε*,^* may have undergone S_1_ → T_1_ ISC with Δ*E*^S_1_/T_1_^ = −0.25 eV and *k*^ISC^ = 2.87 × 10^5^ s^−1^, followed by T_1_ → S_0_ phosphorescence with Δ*E*^T_1_→S_0_^ = −1.65 eV (*λ*^phos^ = 750 nm) and *k*^phos^ = 1.63 × 10° s^−1^ (Fig. 6a). Although the energy barrier for the variation of *ω* from 1° to 180° (Δ*E*^‡^ = 39.6 kJ mol^−1^) can be overcome by the available S_1_ relaxation energy, the E-[1]^§,*ε*,^* → E-[2]^‡,*ε*,^* → E-[3]^eq,*ε*,^* pathway was found to be kinetically less favorable. To assess the competition between this structural rearrangement and photo deactivation, transition state theory (TST) calculations, described in detail in the (SI) and Table S5, were performed along the corresponding *ω* PES.

At *ε* = 80 and 304 K, the calculated quantized-vibrational rate constant for the *ω* rotation (*k*^Q-vib^ = 7.58 × 10^4^ s^−1^) is approximately 340 times slower than the ISC rate (*k*^ISC^ = 2.59 × 10^7^ s^−1^). This substantial difference indicates that ISC is kinetically favored over the large-amplitude *ω* rotation along the relaxation pathway. Therefore, the photo deactivation processes are kinetically favored within the Franck–Condon region (*ω* ≈ 1°), preceding the large-amplitude *ω* rotation. A similar trend was observed at *ε* = 1 (Fig. 5b), where the same planar structure featuring the O5–H5 → O4 ESIPT was identified in the S_1_ state, albeit with reduced fluorescence and phosphorescence rates (Fig. 6b), along with a significantly higher energy barrier (Δ*E*^‡^ = 50.6 kJ mol^−1^) for the E-[1]^§,^* → E-[2]^‡,^* → E-[3]^eq,^* pathway.

To examine the effects of excited-state dynamics on the proposed deactivation processes, microcanonical-ensemble (NVE) molecular-dynamics surface-hopping (MDSH) simulations were performed in the gas phase using 60 Wigner-sampled initial configurations for S_0_ → S_1_ vertical excitation;^46^ the simulation protocol and representative results are summarized in Fig. S3. The trajectories initiated near the Franck–Condon region reveal transient proton-transfer dynamics associated with ESIPT, characterized by back-and-forth proton shuttling within the O5–H5⋯O4 intramolecular hydrogen bond roughly within 98–140 fs after excitation, as monitored through the time evolution of the O5–H5 distance. During these ultrafast proton-transfer dynamics, the *ω* dihedral angle undergoes only relatively small fluctuations. Despite the relatively high kinetic energy generated during excited-state relaxation, *ω* does not approach or exceed the ∼90° torsional configuration identified from the static PES analysis. Following S_1_→S_0_ surface hopping, the system instead relaxes toward LU-C5, the equilibrium structure on the S_0_ PES. These dynamic results support the static PES/TST mechanism and confirm that the ultrafast proton-transfer dynamics and electronic deactivation processes precede the large-amplitude *ω* torsional relaxation.

### p*K*_a_ and 
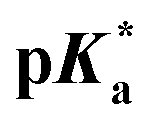
 of LU

#### Born–Haber cycle model

The p*K*_a_ and 
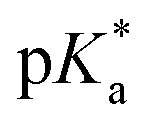
 values corresponding to the deprotonation sites of LU, calculated using the Born–Haber cycle in the S_0_ and S_1_ states, without and with an explicit water molecule, are presented in Table 1, along with available theoretical and experimental data. These results are further compared in Fig. 7a and b. Notably, the p*K*_a_ of LU obtained from spectrophotometric measurements varied across a moderately wide range, ranging from ∼6.9 to ∼10.3^21^; additionally, p*K*_a_ predictions based on the Born–Haber cycle were highly sensitive to the choice of experimental Gibbs free energies associated with the proton in both the gas phase 
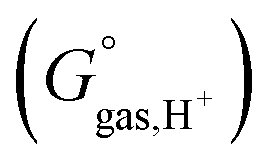
 and aqueous solution 
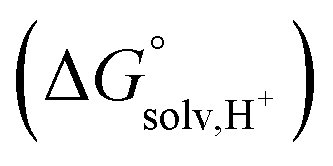
; the reported values for 
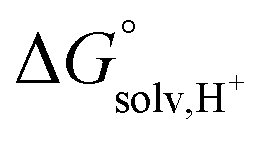
 significantly varied in the literature, ranging from −1085.7 to −1104.6 kJ mol^−1^.^38^

p*K*_a_ and 
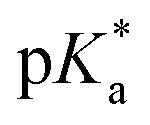
 obtained from the Born–Haber cycle model and DFT and TD-DFT/B3LYP/aug-cc-pVDZ methods, respectively, along with reported theoretical and experimental values. All the excited state equilibrium structures of the neutral forms are characterized by LU-C4 with ESIPT. Notations and symbols are defined in Table S1

**Table:** 

	p*K*^a^_a_ DFT/B3LYP/aug-cc-pVDZ		p*K*^b^_a_ DFT/B3LYP/6-311+G*	p*K*_a_ experiment
	LU	LU–H_2_O	LU	
LU-C5	16.1	13.8	14.1	10.8^d^ (pH ∼ 12)
LU-C3′	13.2	11.0	12.9	9.3^d^, 10.3^c^ (pH ∼ 9)
LU-C7	9.4	8.2	9.3	7.3^d^, 8.6^c^ (pH ∼ 7)
LU-C4′	6.9	6.3	7.5	8.5^d^, 6.9^c^ (pH < 5)

**Table:** 

	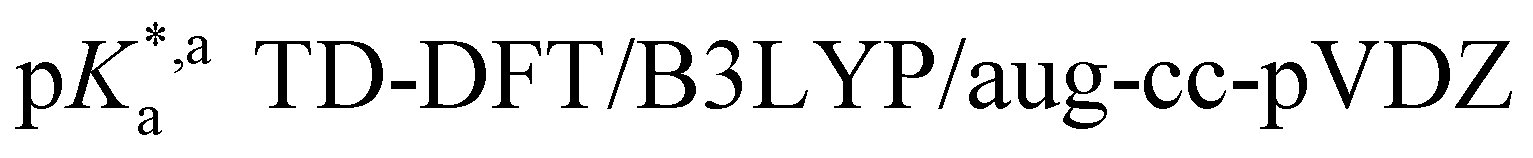	
	LU	LU–H_2_O
LU-C4	19.1	12.5
LU-C3′	6.8	7.4
LU-C7	10.3	8.7
LU-C4′	5.1	5.6

**Fig. 7:**
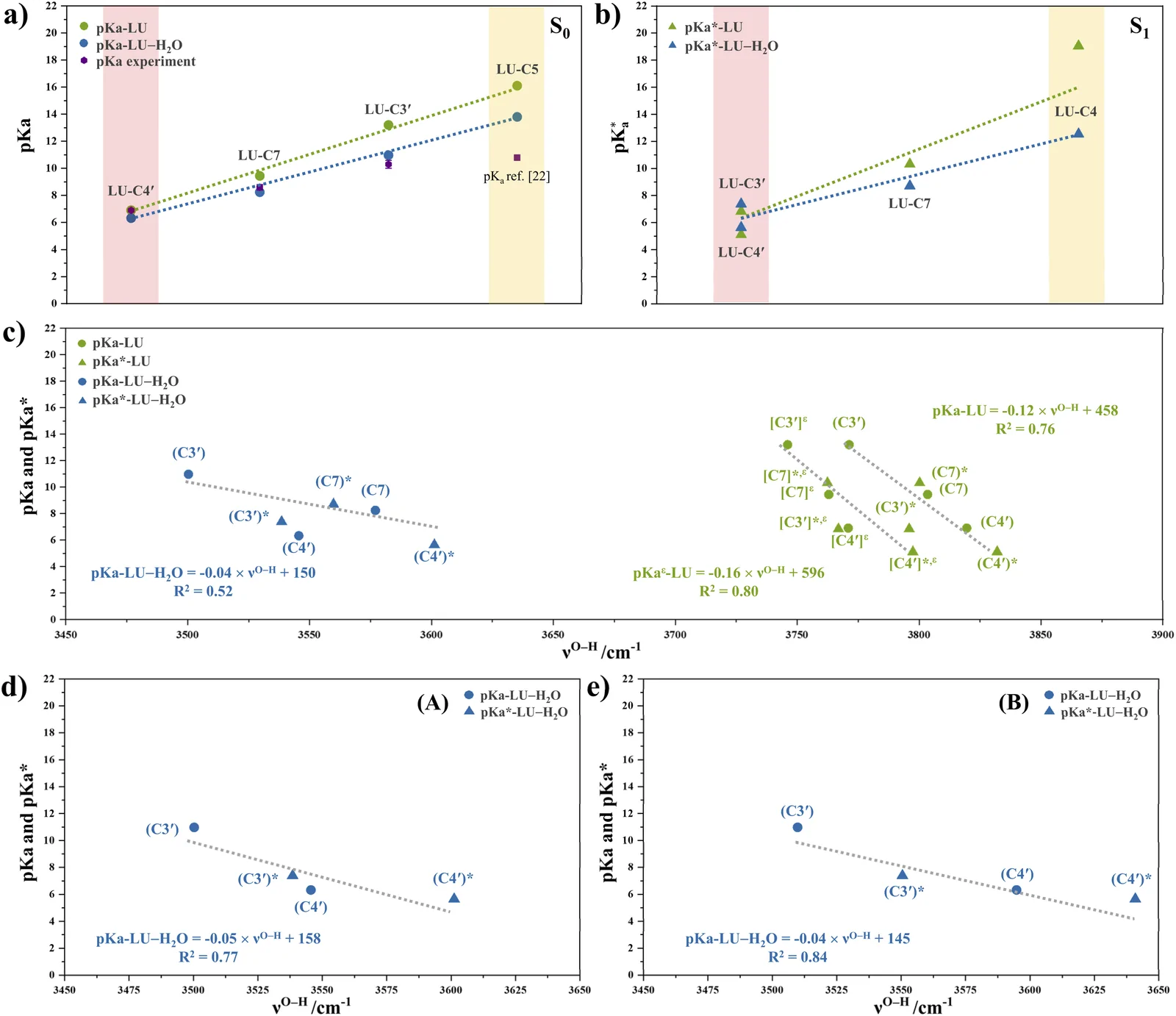
(a) and (b) The acidity constants corresponding to the deprotonation sites of LU, calculated using the Born–Haber cycle in the S_0_ and S_1_ states, with implicit and explicit water models, respectively, compared with available experimental data. (c) Correlations between the acidity constants obtained from the Born–Haber cycle model and O–H stretching frequencies at the deprotonation sites of LU in the S_0_ and S_1_ states, computed based on the isolated LU, implicit (*ε* = 80) and explicit water (1 : 1 LU–H_2_O complex) models. (d) and (e) Correlations between the acidity constants obtained from the Born–Haber cycle model and O–H stretching frequencies at LU-C3′ and LU-C4′, in the S_0_ and S_1_ states, obtained based the explicit water (1 : 1 LU–H_2_O complexes) model, with (A) and without (B) the O5–H5 → O4 intramolecular H-bond and ESIPT, respectively. (…) = Calculations with *ε* = 1; […]^*ε*^ = implicit water model with *ε* = 80. * = Value obtained in the excited electronic state.

Herein, we intended to determine an appropriate value of 
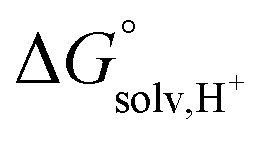
 for proton dissociation from the O–H groups of the catechol moiety by calibrating against the reported experimental p*K*_a_ of the most acidic site (LU-C4′) in the S_0_ state. Within the implicit solvation model, the Gibbs free energy of the proton in aqueous solution was systematically varied over the range 
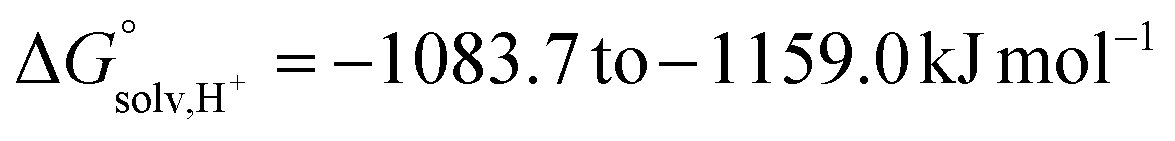
. The results showed that 
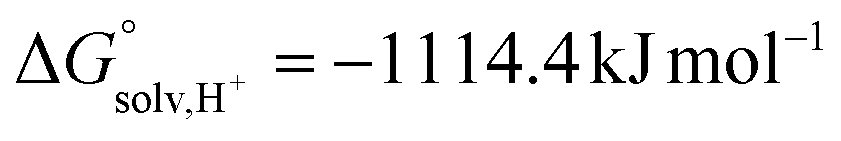
 reproduced the experimental p*K*_a_ of 6.9. This optimized value agreed well with the recommended literature value (
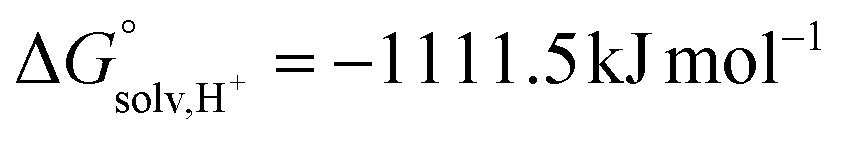
, ref. 47). Using this value, the p*K*_a_ values for LU-C7, LU-C3′, and LU-C5 were calculated as 9.4, 13.2, and 16.1, respectively (Table 1).

For the explicit solvation model (1 : 1 LU–H_2_O H-bond complexes), the optimized 
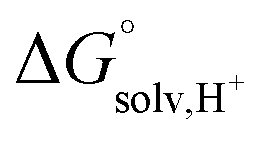
 yielded p*K*_a_ values of 6.3, 8.2, 11.0, and 13.8 for LU-C4′, LU-C7, LU-C3′, and LU-C5, respectively. The calculated site-specific p*K*_a_ values can be compared most directly with the experimental first acid dissociation constant of neutral LU, p*K*_a_ = 6.9 ± 0.1, measured spectroscopically in methanol–water (1 : 2 v/v).^21^ The higher experimental values, p*K*_a_ = 8.6 ± 0.2 (pH ≈ 7) and 10.3 ± 0.3 (pH ≈ 9), correspond to subsequent deprotonations of the mono- and dianionic forms, respectively,^21^ while a value of p*K*_a_ = 10.8 (pH ≈ 12) has been reported for further deprotonation of the trianionic form.^22^

These sequential macroscopic p*K*_a_ values should not be directly assigned to the calculated site-specific p*K*_a_ values because the present calculations consider alternative single-site deprotonations of neutral LU rather than the successive deprotonations of the corresponding charged microstates. Accordingly, the Born–Haber calculations in the S_0_ state are primarily used here to establish the relative site-specific acidities and identify the preferred first deprotonation site. The O–H group at LU-C4′ on the catechol ring exhibits the highest acidity, whereas that at LU-C3′ is less acidic. On the resorcinol ring, the O–H group at LU-C7 is weakly acidic, while that at LU-C5 is least prone to proton dissociation because of the strong intramolecular O5–H5⋯O4 H-bond; instead, O4 preferentially acts as a proton acceptor toward a water molecule (Fig. S1b).

Assuming that the aqueous solvent is not directly involved in the photoexcitation process, the optimized value of 
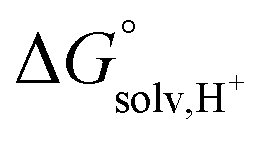
 was further employed in the 
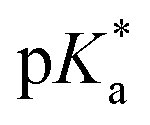
 calculations using the Born–Haber cycle model. The results presented in Table 1 and Fig. 7b indicate that in the S_1_ state, for LU-C4′, LU-C7, LU-C3′, and LU-C5, the implicit solvation model yielded 
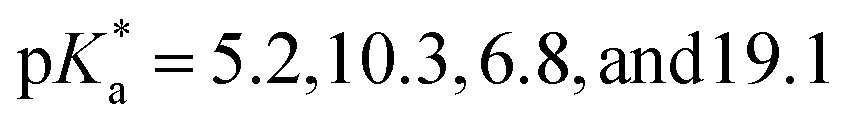
, respectively, whereas the explicit solvation model yielded 
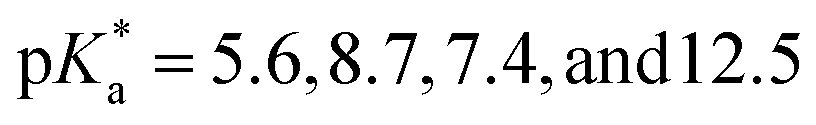
. Notably, in both the implicit and explicit solvation models, the O–H group on the resorcinol ring (LU-C5) remained basic in the S_1_ state, whereas those on the catechol ring became more acidic. Specifically, the O–H group at LU-C3′ became weakly acidic in the implicit solvation model 
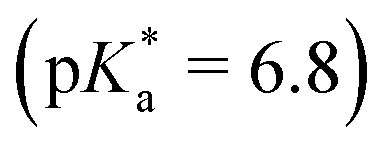
.

Detailed analysis of the Gibbs free energy changes presented in Table S3 indicated that the acidity of each deprotonation site in both the S_0_ and S_1_ states correlated with the Gibbs free energy change associated with solvation of the deprotonated form of LU 
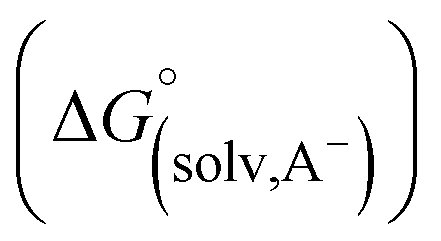
 in Fig. 2b). In particular, lower values of 
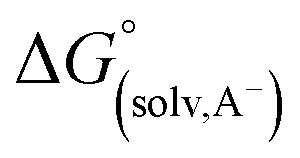
 corresponded to higher acidities (Table S3). For example, within the implicit solvation model in the S_1_ state, LU-C4 exhibited the lowest acidity 
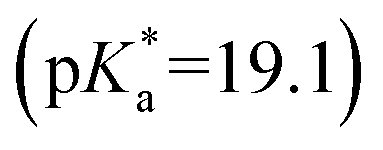
 among all deprotonation sites. The high p*K*_a_ and 
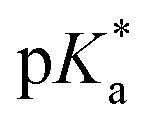
 values in the S_0_ and S_1_ states, respectively, were attributed to the intrinsic electrostatic repulsion between adjacent partial negative charges on the O5 and O4 atoms after deprotonation.

#### Vibrational frequency – p*K*_a_ correlation model

All the vibrational spectra of LU in the ground and excited electronic states in the gas phase and in continuum aqueous solutions are include in Fig. S4. The O–H stretching frequencies (*ν*^O−H^) of the deprotonation sites of LU in the S_0_ and S_1_ states, obtained using the three computational models, are presented in Table S6. Because the strong intramolecular O5–H5⋯O4 H-bond suppresses proton dissociation from the O5–H5 group toward water, while O4 preferentially acts as a H-bond acceptor toward water (Fig. S1), its p*K*_a_, 
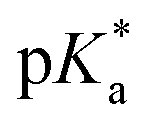
, and *ν*^O–H^ values were excluded from the regression analysis.

The correlations among p*K*_a_, 
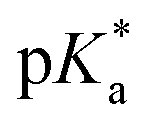
, and *ν*^O–H^ obtained in the gas-phase model (*ε* = 1), implicit solvation model (*ε* = 80), and explicit solvation model (1 : 1 LU–H_2_O complexes) are shown in Fig. 7c. In general, although the *ν*^O–H^ calculated using these three models spanned different ranges, showing a systematic redshift from the isolated to the implicit and explicit solvation models, the vibrational frequency – p*K*_a_ correlations exhibited consistent trends and corroborated the results obtained from the Born–Haber cycle.

In all models, approximate linear relationships were observed between the O–H stretching frequencies and both p*K*_a_ and 
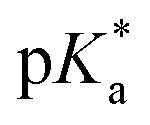
, indicating that the linear correlation between the vibrational frequency and p*K*_a_ was preserved across electronic states. In the gas phase at *ε* = 1, the correlations in the S_0_ and S_1_ states exhibited a linear relationship; p*K*_a_ = −0.12 × *ν*^O–H^ + 458 (*R*^2^ = 0.76), whereas at *ε* = 80, p*K*_a_ = −0.16 × *ν*^O–H^ + 596 (*R*^2^ = 0.80). A similar linear correlation (although relatively weak), *i.e.*, p*K*_a_ = −0.04 × *ν*^O–H^ + 149 (*R*^2^ = 0.52), was observed for the explicit solvation model. It appears that, in all models, photoexcitation induces systematic blue shifts in the *ν*^O–H^ stretching frequencies of the catecholic O–H groups (LU-C4′ and LU-C3′), whereas the O–H group on the benzopyran ring (LU-C7) exhibits a red shift, particularly in the 1 : 1 LU–H_2_O complexes. These contrasting trends indicate fundamentally different photoinduced responses of the catecholic O–H groups and the benzopyran-ring O–H group. The observed behavior suggests that the local electronic and H-bonding environments of LU-C4′/LU-C3′ and LU-C7 are affected differently upon photoexcitation.

Consequently, excluding the *ν*^O–H^ value of LU-C7 from the regression analysis substantially improves the linear relationship for the 1 : 1 LU–H_2_O complexes, yielding 
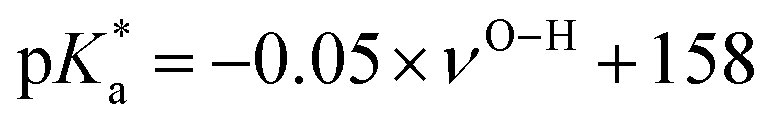
 with *R*^2^ = 0.77 (Fig. 7d). Based on these findings, the influence of intramolecular H-bonding and ESIPT at LU-C5 on the *ν*^O–H^ stretching frequencies at LU-C4′ and LU-C3′ was further investigated by calculating the *ν*^O–H^ values for these O–H groups after removing the O5–H5⋯O4 intramolecular H-bond. As shown in Fig. 7e, the *ν*^O–H^ frequencies at LU-C3′ remain unchanged, whereas those at LU-C4′ exhibit systematic blue shifts. These results indicate that the absence of the O5–H5⋯O4 intramolecular H-bond has a negligible effect on the vibrational behavior of LU-C3′ but significantly influences that of LU-C4′.

To further examine whether separate vibrational frequency-acidity correlations can be established for p*K*_a_ and 
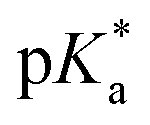
, regression analyses were performed independently for the ground-state p*K*_a_ and excited-state 
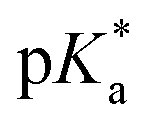
 datasets and compared with the combined-state correlations. For both the isolated LU and implicit-solvation models, excellent correlations were obtained for the ground-state p*K*_a_ data (*R*^2^ = 0.99; Fig. S5). For the excited-state 
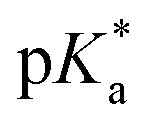
 data, the implicit-solvation model retained a reasonable near-linear correlation (*R*^2^ = 0.68), whereas a weaker correlation was obtained for isolated LU (*R*^2^ = 0.46). These results suggest that separate *ν*^O–H^–p*K*_a_ and 
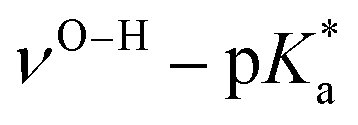
 relationships can be identified, although their strengths depend on the electronic state and solvation model.

It should be emphasized that p*K*_a_ and 
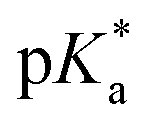
 are solution-phase equilibrium quantities; therefore, correlations obtained for isolated LU without a representation of solvent effects should be regarded primarily as reference calculations rather than direct models of experimental acidity. In this respect, inclusion of solvent effects, at least approximately through a dielectric continuum, provides a more physically relevant description of the p*K*_a_–*ν*^O–H^ and 
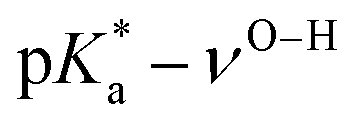
 relationships. Separate regressions were not considered for the explicit-solvation model because only two data points would remain for each electronic state, precluding a meaningful statistical analysis. These results indicate that the combined 
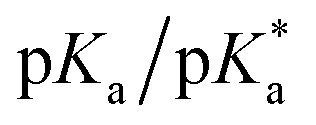
 correlations represent an approximate cross-state trend rather than a universal linear relationship.

#### Förster cycle model

Because the *R*^2^ value for the implicit solvation model was higher than that of the isolated LU model (Fig. 7c), only the 
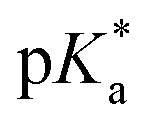
 values obtained at *ε* = 80 were considered for comparison with those derived from the Förster cycle model. Based on the Gibbs free energies for proton dissociation in the S_1_ and S_0_ states (Δ*G*_diss_ and 
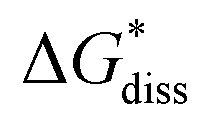
, respectively), the 
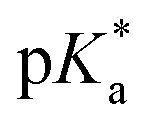
 values at 298 K for all the deprotonation sites were calculated and presented in Table 2. The 
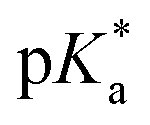
 values obtained from the Förster cycle model, using both the implicit (*ε* = 80) and explicit solvation models, were compared well with those derived from the Born–Haber cycle model (Fig. 8).

**Table 2:** (a) and (b) 
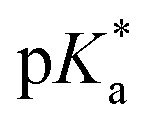
 of LU and LU–H_2_O obtained from the Förster cycle model and TD-DFT/B3LYP/aug-cc-pVDZ methods with implicit (*ε* = 80) and explicit water (1 : 1 LU–H_2_O complexes) models, respectively. All the excited state equilibrium structures of the neutral forms are characterized by LU-C4 with ESIPT. Notations and symbols are defined in Table S1

	p*K*_a_^a^	Δ*G*_diss_	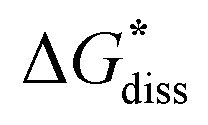	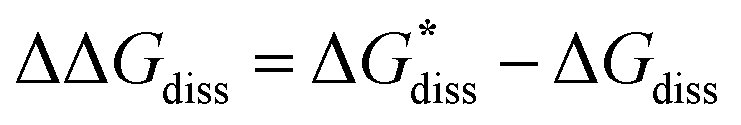	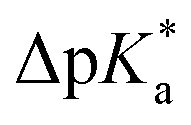	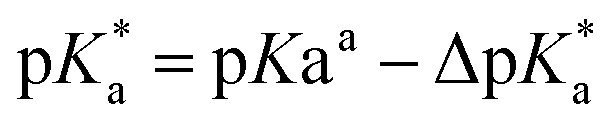
**(a)LU**						
LU-C5	16.1	1232.61	1249.87	−17.26	−3.02	19.1
LU-C3′	13.2	1215.84	1182.31	33.52	5.87	7.3
LU-C7	9.4	1194.57	1188.35	6.22	1.09	8.3
LU-C4′	6.9	1180.05	1170.28	9.77	1.71	5.2

**(b) LU–H**_**2**_ **O**						
LU-C5	13.8	1219.42	1212.23	7.19	1.26	12.5
LU-C3′	11.0	1203.26	1182.77	20.49	3.59	7.4
LU-C7	8.2	1187.67	1190.36	−2.69	−0.47	8.7
LU-C4′	6.3	1176.99	1172.30	4.69	0.82	5.5

**Fig. 8:**
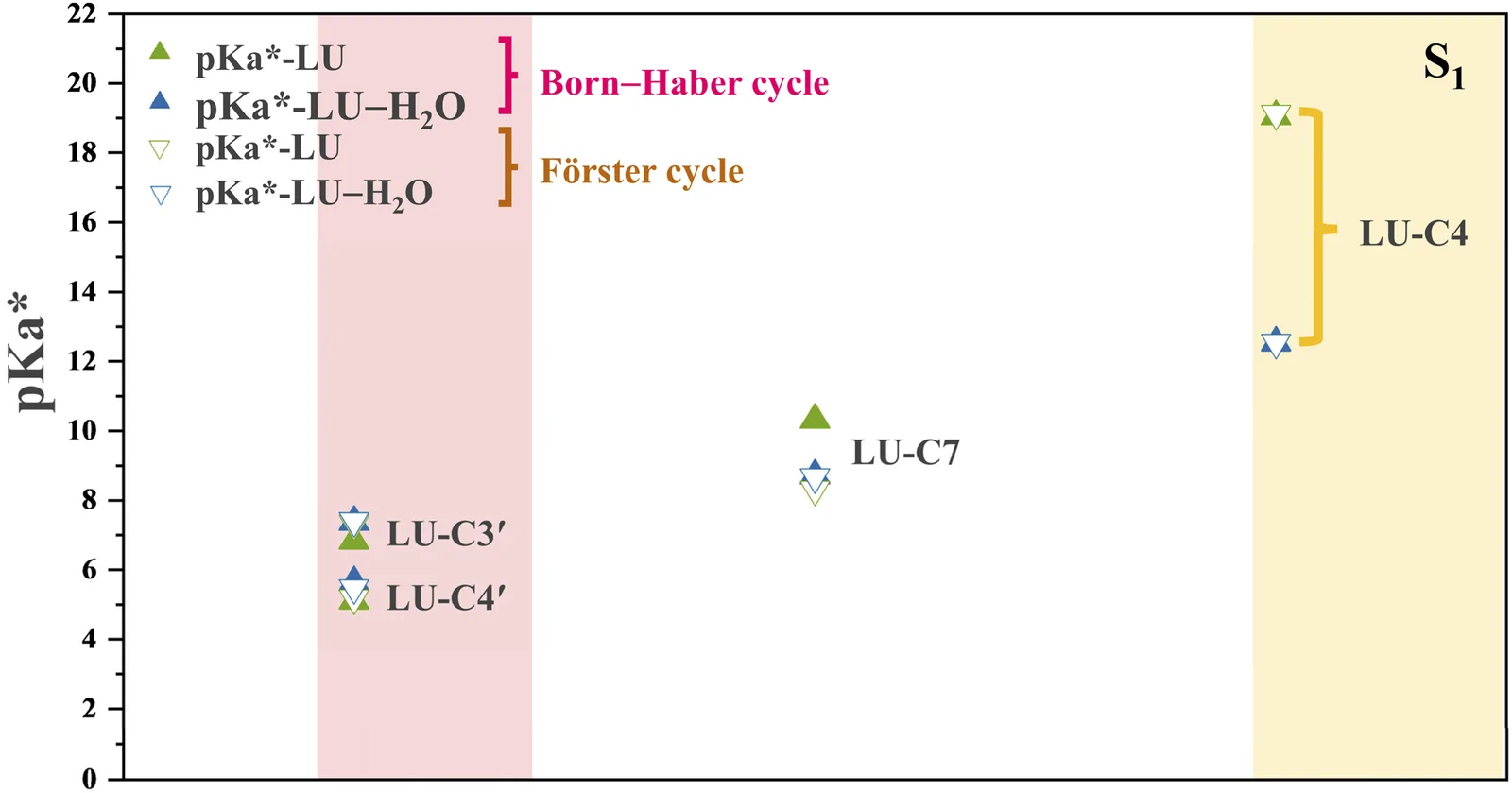
Comparison of the 
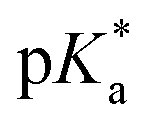
 values obtained using the Born–Haber and Förster cycle models. p*K*_a_-LU and p*K*_a_-LU–H_2_O = acidity constants obtained based on implicit (*ε* = 80) and explicit water (1 : 1 LU–H_2_O complex) models in the S_0_ state, respectively; 
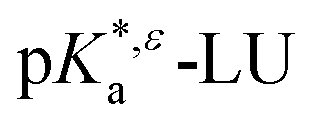
 and 
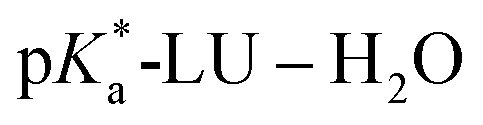
 = acidity constants obtained based on implicit (*ε* = 80) and explicit water (1 : 1 LU–H_2_O complex) models in the S_1_ state, respectively.

Although the Born–Haber and Förster-cycle approaches are thermodynamically related, they involve different sets of calculated quantities: the Born–Haber approach determines p*K*_a_ and 
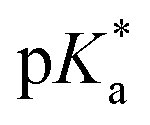
 separately using the Gibbs free energies of all neutral and deprotonated equilibrium structures involved in the respective thermodynamic cycles, whereas the Förster cycle determines 
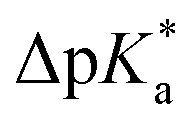
 from two equilibrium structures (ArOH^eq^ and ArO^−,eq,^* in Fig. 2c) and the corresponding vertical S_0_ → S_1_ absorption and S_1_ → S_0_ emission energies, requiring only an accurate ground-state p*K*_a_, obtained either from the Born–Haber cycle or experiment, to derive 
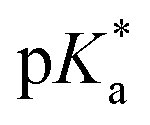
. The close agreement between the resulting 
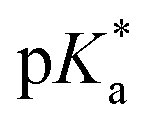
 values therefore demonstrates the internal consistency of the two thermodynamically related approaches rather than their independent validation. Taken together, the Born–Haber cycle, vibrational frequency – 
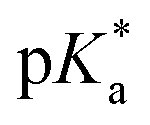
 correlations, and Förster-cycle analyses provide complementary perspectives on the site-specific ground- and excited-state acidities of LU.

Overall, the three proposed complementary models provide different but interconnected perspectives on the ground- and excited-state acidities of LU. The Born–Haber cycle provides direct thermodynamic estimates of the site-specific p*K*_a_ and 
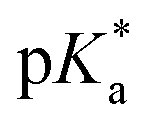
, whereas the *ν*^O–H^–p*K*_a_ relationships connect the calculated acidities with the local vibrational characteristics of the O–H groups. The Förster cycle provides an alternative route to 
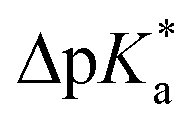
 through the corresponding absorption and emission energies.

## Conclusions

The ESIPT mechanisms, photoacidity, and photophysical properties of LU, a multifunctional molecule with several potential deprotonation sites, were systematically investigated using validated computational approaches. The study aimed at providing detailed insight into the structural, energetic, and photophysical factors governing the excited-state acidity, with particular emphasis on ESIPT mediated by hydrogen bonding across environments with dielectric constants *ε* ranging from 1 to 80.

Benchmarking against available theoretical and experimental data confirmed the reliability of the employed DFT and TD-DFT/B3LYP/aug-cc-pVDZ methods. The results indicated that LU adopted a planar equilibrium geometry in both the S_0_ and S_1_ states. Key radiative and nonradiative decay pathways—including ultrafast ESIPT, fluorescence, ISC, and phosphorescence—predominantly occurred within the Franck–Condon region (*ω* ≈ 0°–1°). In contrast, rotation of the dihedral angle between the benzopyran and catechol moieties toward ∼90° reduced π-conjugation and led to distinct electron density distributions, specifically under varying dielectric conditions.

The acidity constants in both the ground and excited states (p*K*_a_ and 
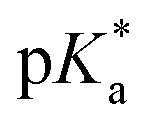
, respectively) were evaluated using three complementary models: (i) a gas-phase model using isolated LU; (ii) an implicit solvation model; and (iii) an explicit solvation model involving 1 : 1 LU–H_2_O H-bonded complexes at the LU-C5, LU-C7, LU-C3′, and LU-C4′ positions. Ground-state results based on the Born–Haber cycle model revealed that the O–H group at LU-C4′ exhibited the highest acidity on the catechol ring (p*K*_a_ ≈ 6–7), whereas LU-C3′ behaved as a weak base (p*K*_a_ ≈ 11–13). On the resorcinol ring, LU-C7 showed moderate basicity (p*K*_a_ ≈ 8–9), while LU-C5 was not prone to deprotonation (p*K*_a_ ≈ 14–16) owing to a stabilizing intramolecular H-bond.

In the excited state, a redistribution of acidity was observed. The LU-C4′ site became more acidic 
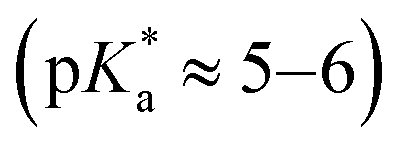
, and LU-C3′ transitioned into a weak acid 
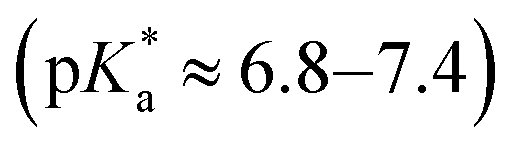
. Conversely, deprotonation at LU-C7 and LU-C5 became less favorable 
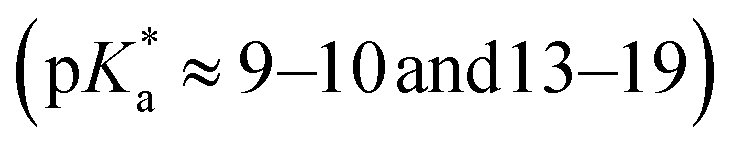
, respectively). Analysis of Gibbs free energy changes showed that the acidity of each deprotonation site in both the S_0_ and S_1_ states correlated with the solvation free energy of the deprotonated LU species in solution, where lower 
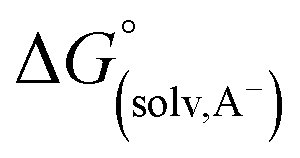
 values (higher stabilization in solution) indicated higher acidity.

The DFT and TD-DFT calculations reveal that the intramolecular H-bond and the associated ESIPT involving the benzopyran-ring O–H group at LU-C5 directly influence the vibrational properties and acidity of the most acidic catecholic O–H group at LU-C4′. Despite differences in absolute O–H stretching frequencies between the implicit and explicit solvation models, consistent vibrational frequency – p*K*_a_ correlations were observed. In all cases, near–linear relationships were observed between O–H stretching frequencies and both p*K*_a_ and 
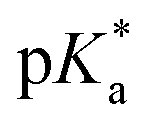
, demonstrating that these correlations persisted across solvation environments and electronic states; stronger blue shifts of the O–H stretching frequencies in the excited state corresponded to stronger photoacidity.

It should be emphasized that although the present results reveal a near–linear relationship between the calculated p*K*_a_ and 
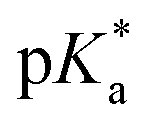
 values and the local O–H stretching frequencies for LU, this relationship should not be regarded as universal. Its quantitative form is expected to depend on the molecular structure, substituent effects, solvation environment, and experimental conditions.

Excited-state acidity constants derived from the Förster cycle were in close agreement with those obtained from the Born–Haber approach, indicating the state-function nature of the associated Gibbs free energies. The combined application of these three models thus provides thermodynamic, vibrational, and photophysical insights into the site-specific acidities of LU in the S_0_ and S_1_ states.

Based on the present finding that LU-C4′ is the most acidic site in both the ground and excited electronic states, further theoretical and experimental investigations could explore strategies to enhance its acidity while preserving the C4′–OH group. Molecular engineering through selective functionalization of the other O–H sites, including the introduction of phenyl-containing substituents and subsequent lactose conjugation, could also be investigated to achieve desirable photophysical properties, particularly absorption in the visible/NIR region, while optimizing the hydrophilic/hydrophobic balance required for an effective delivery system. Such molecular engineering may further enhance the potential of LU for medical and pharmaceutical applications.

Overall, this study provides a comprehensive molecular-level understanding of excited-state deprotonation in LU. The insights gained into its electronic structure, energetics, and photophysical behavior offer valuable guidance for the rational design and optimization of LU-based compounds with improved functional properties, with potential applications in pharmacology, medicine, nutraceuticals, and drug development.

## Author contributions

Photiganit: methodology; data curation; formal analysis; investigation; software; validation; visualization. Thongyu: methodology; investigation; software; validation; visualization. Panajapo: methodology; investigation; software. Thisuwan: funding acquisition; project administration; methodology; data curation; formal analysis; investigation; software; writing – original draft; writing – review & editing. Sagarik: supervision; project administration; conceptualization; writing – original draft; writing – review and editing.

## Conflicts of interest

All of the authors declare no conflict of interest.

## Supplementary Material

RA-016-D6RA05576A-s001

## Data Availability

All data generated or analyzed during this study are included in this published article and its additional supplementary information (SI) files. Supplementary information: equilibrium geometries, NTO and energetic of the key-structues on the PESs, thermodynamic properties and NVE-MDSH results. See DOI: https://doi.org/10.1039/d6ra05576a.
