# Supplementary material for: Photoacidity and ESIPT in luteolin: acidity-vibrational frequency relationships across electronic states
Source: RSC Adv. 2026 Sep 24;16(45):57274–87. doi: 10.1039/d6ra05576a (PMC13607960; doi:10.1039/d6ra05576a)
Supplement: Supplementary file 1 [file RA-016-D6RA05576A-s001.pdf]

Supplementary Information (SI) for RSC Advances.
This journal is © The Royal Society of Chemistry

Electronic Supplementary Information (ESI)

Photoacidity and ESIPT in Luteolin: Acidity−Vibrational Frequency Relationships Across Electronic States

By

Siramol Photiganit[a]
Ananda Thongyu[a]
Pannipa Panajapo[a]
Jittima Thisuwan[b,*]
and
Kritsana Sagarik,[a,*]

[a)]*School of Chemistry, Institute of Science, Suranaree University of Technology, Nakhon Ratchasima 30000, Thailand.*
[b)]*Division of Science, Faculty of Education, Nakhon Phanom University, Nakhon Phanom 48000, Thailand.*

*Corresponding author: *kritsana@sut.ac.th, thisuwan@gmail.com*
[†]Electronic Supplementary Information (ESI) available
Keywords: Luteolin, photoacidity, DFT and TD-DFT, Born-Haber cycle, Förster cycle, ESIPT.

Figure S1 a)–b) Gas-phase equilibrium structures, total and Gibbs free energies of the 1:1 and 1:4 LU–H_2O complexes in the S_0 and S_1 states, respectively. (A) and (B) denote structures with and without the O5–H5→O4 intramolecular H–bond and ESIPT, respectively. E^{Tot} = total energy in au; $\Delta E^{S_0 \rightarrow S_1}$ = vertical excitation energy in eV; λ^{abs} = absorption wavelength in nm; […] = value obtained in ε = 80. (A) and (B) = structures with and without the O5–H5→O4 intramolecular H–bond and ESIPT, respectively.

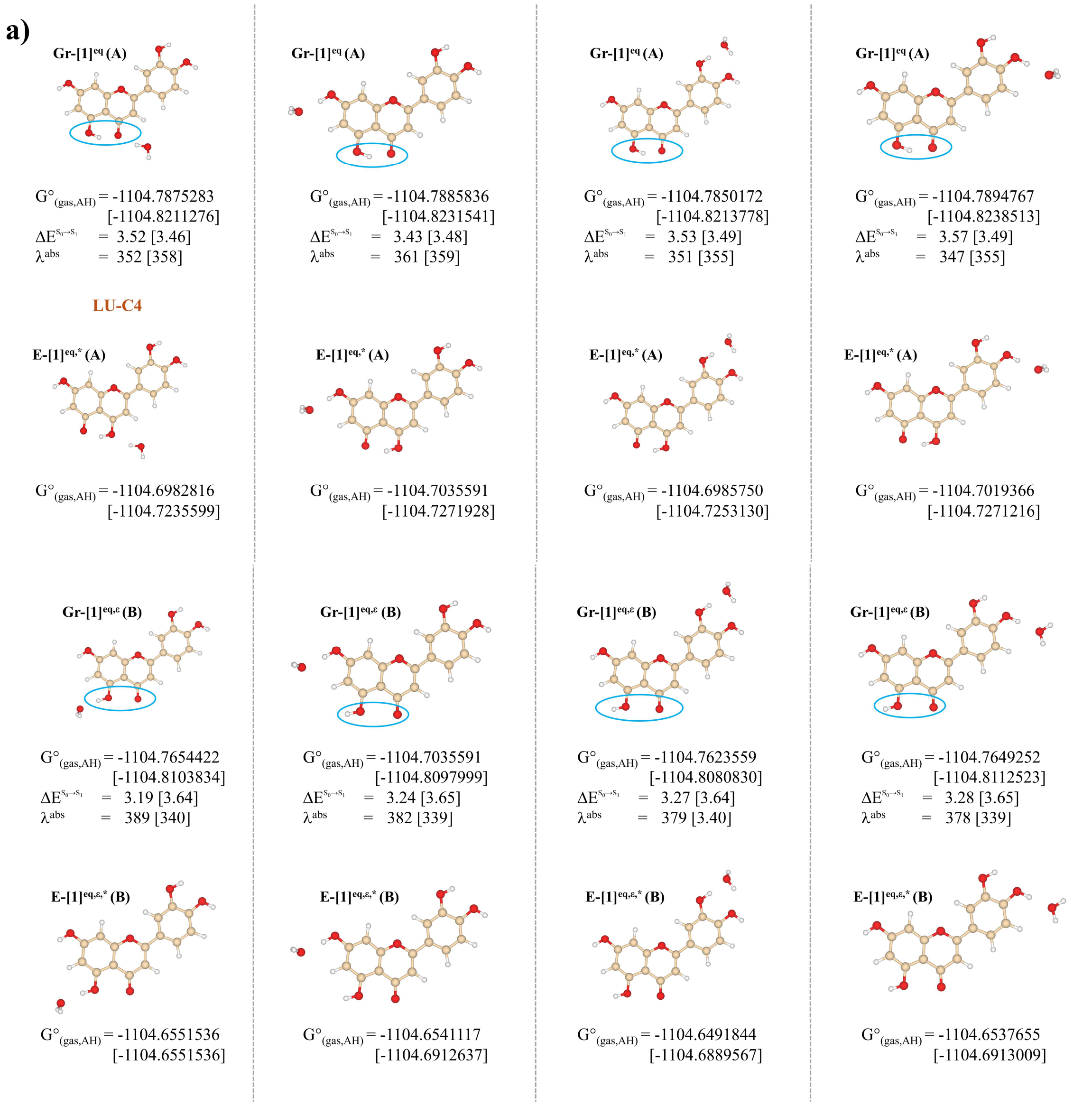

Figure S1 (Cont.)

b)

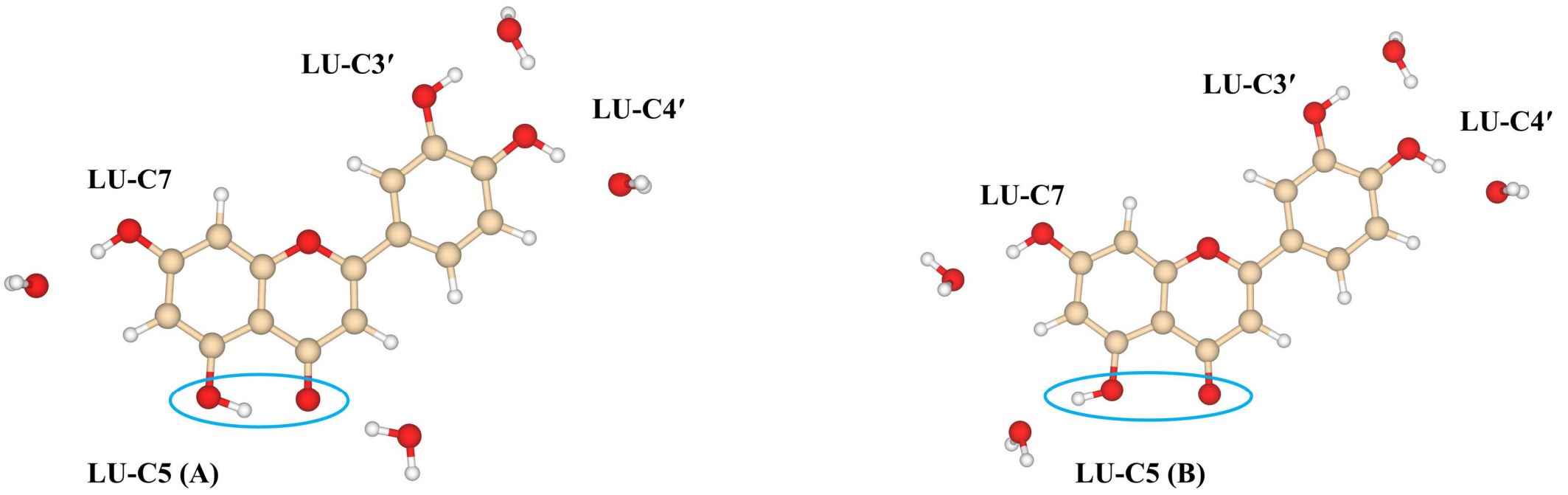

E^{Tot} = -1334.2474714	E^{Tot} = -1334.2206149
$\Delta E^{S_0 \rightarrow S_1}$ = 3.48	$\Delta E^{S_0 \rightarrow S_1}$ = 3.45
λ^{abs} = 356	λ^{abs} = 359

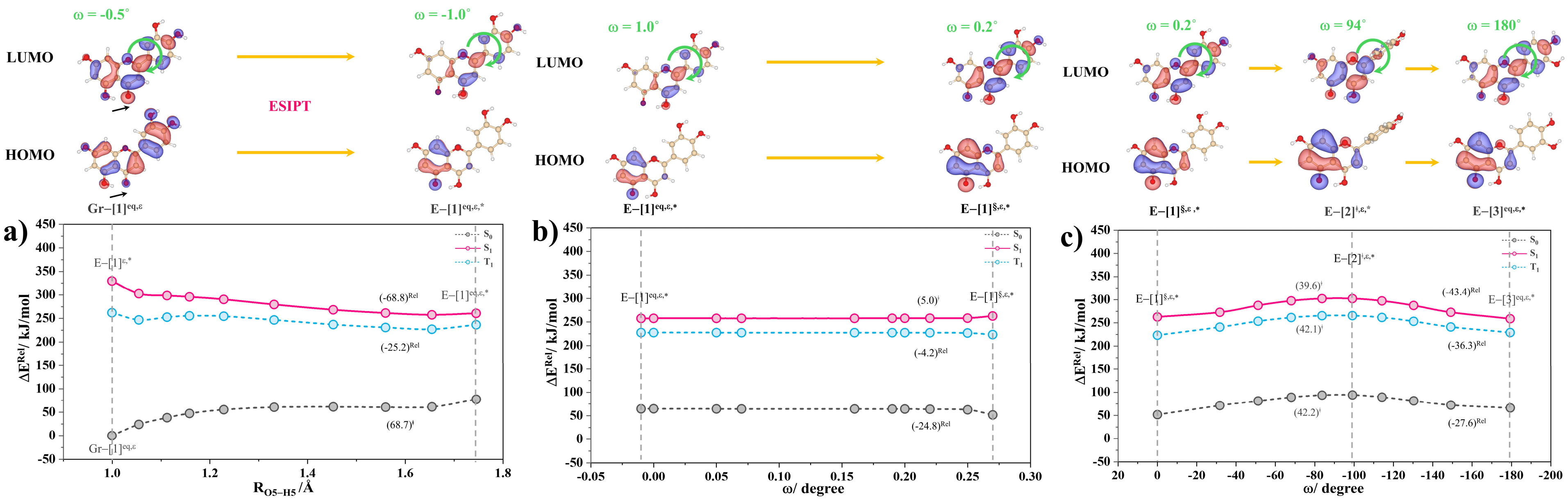

Figure S2 Potential energy surfaces (PESs) for the structural relaxation of LU in the S_1 state at ε = 80. a) PES along the O5–H5→O4 proton-transfer coordinate, with the O5–H5 distance (R_{O5-H5}) varied from 1.0 to 1.7 Å, showing the barrierless ESIPT from O5 to O4, and leading to the proton-transferred LU-C4 structure. b) PES for the small-amplitude variation of the dihedral angle ω from approximately 0 to 0.27° within the Franck–Condon region. c) PES for the large-amplitude rotation of ω from 0 to 180°. Notations and symbols are defined in Table S1†. ΔE^{Rel} = relative energy with respect to the precursor in the S_0 state in kJ/mol; $(\ldots)^{Rel}$ and $(\ldots)^{‡}$ = relaxation energy and energy barrier on the PES in kJ/mol.

Figure S3 a)–d) Representative results on the time evolutions of the total energies, temperature, dihedral angle (ω) and ESIPT measured by the O5–H5 distance, obtained from NVE-MDSH simulations. $\tau_{MDSH}^{S_1 \to S_0}$ = surface hopping time; $\langle T \rangle_{S_1}$ and $\langle T \rangle_{S_0}$ = average temperature in the S_1 and S_0 states; $\langle \omega \rangle_{S_1}$ = average dihedral angle in the S_1 state. ω_{SH} = dihedral angle of the $S_1 \to S_0$ hopping structure.

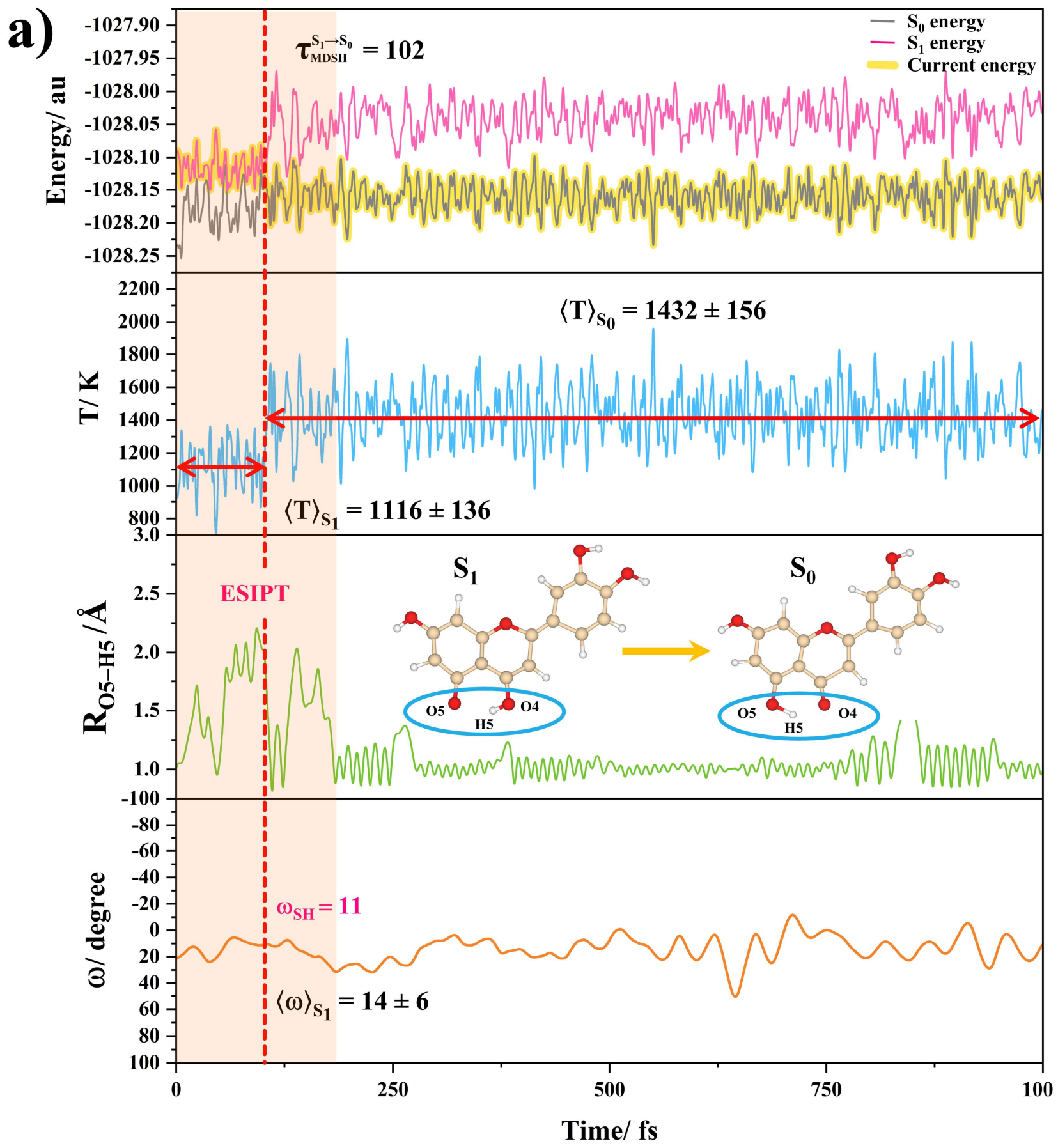

Note: NVE-MDSH simulations were performed using the TURBOMOLE/NEWTON-X interface. Initial nuclear coordinates and momenta were generated from a Wigner distribution around the optimized S_0 equilibrium structure, and 60 configurations were used for $S_0 \to S_1$ vertical excitation. The nonadiabatic trajectories were propagated in the microcanonical (NVE) ensemble with a time step of 0.2 fs for up to 2.5 ps using the surface-hopping algorithm implemented in NEWTON-X,[1] with electronic-structure energies, gradients, and nonadiabatic coupling quantities evaluated using TURBOMOLE[2] with the DFT/B3LYP/aug-cc-pVDZ method.

1. M. Barbatti, M. Ruckenbauer, F. Plasser, J. Pittner, G. Granucci, M. Persico and H. Lischka, *Wiley Interdiscip. Rev.: Comput. Mol. Sci*., 2014, **4,** 26–33.

2. S. G. Balasubramani, G. P. Chen, S. Coriani, M. Diedenhofen, M. S. Frank, Y. J. Franzke, F. Furche, R. Grotjahn, M. E. Harding, C. Hattig, A. Hellweg, B. Helmich-Paris, C. Holzer, U. Huniar, M. Kaupp, A. Marefat Khah, S. Karbalaei Khani, T. Muller, F. Mack, B. D. Nguyen, S. M. Parker, E. Perlt, D. Rappoport, K. Reiter, S. Roy, M. Ruckert, G. Schmitz, M. Sierka, E. Tapavicza, D. P. Tew, C. van Wullen, V. K. Voora, F. Weigend, A. Wodynski and J. M. Yu, *J. Chem. Phys*., 2020, **152,** 184107.

Figure S3 (Cont.)

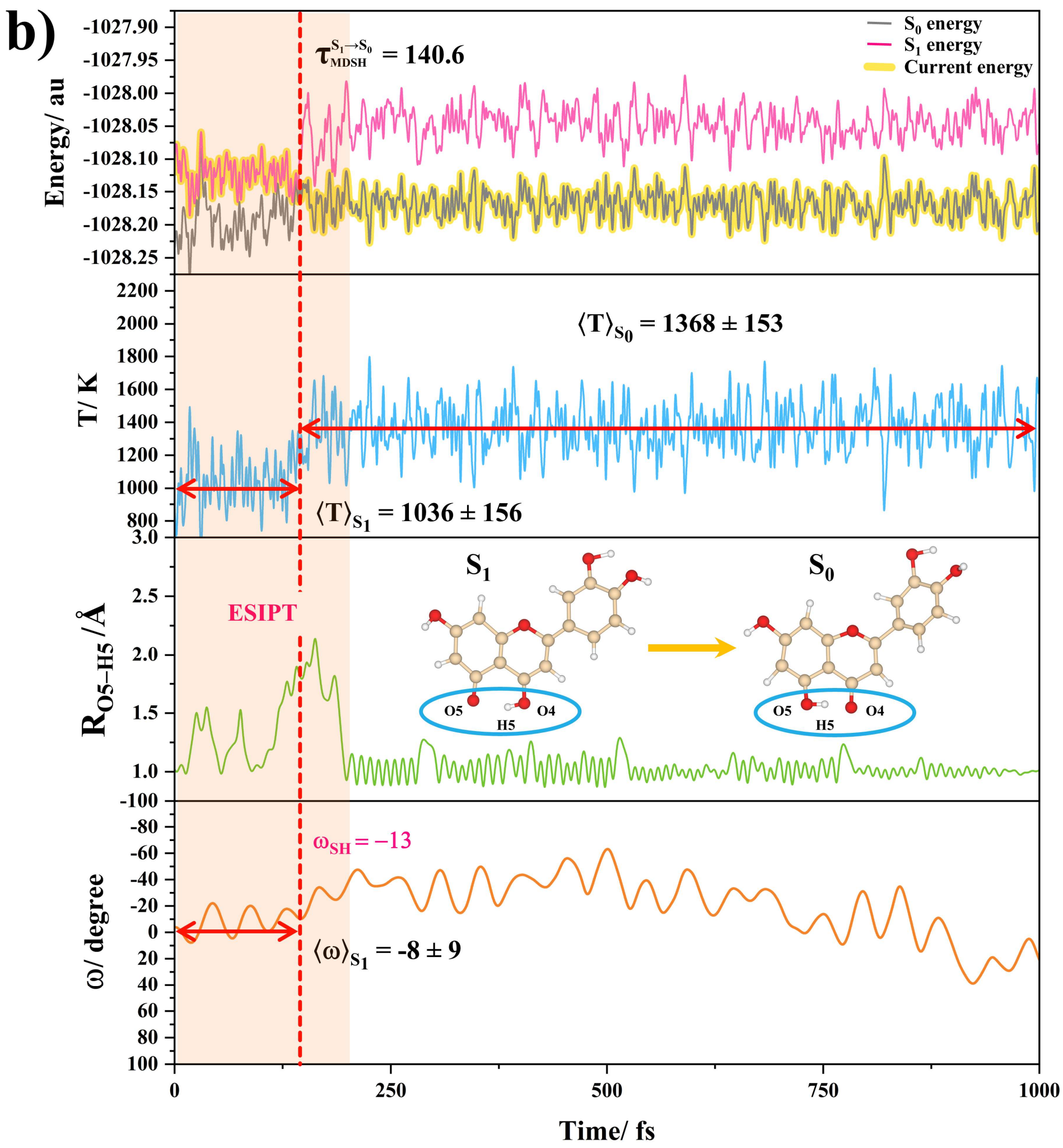

Figure S3 (Cont.)

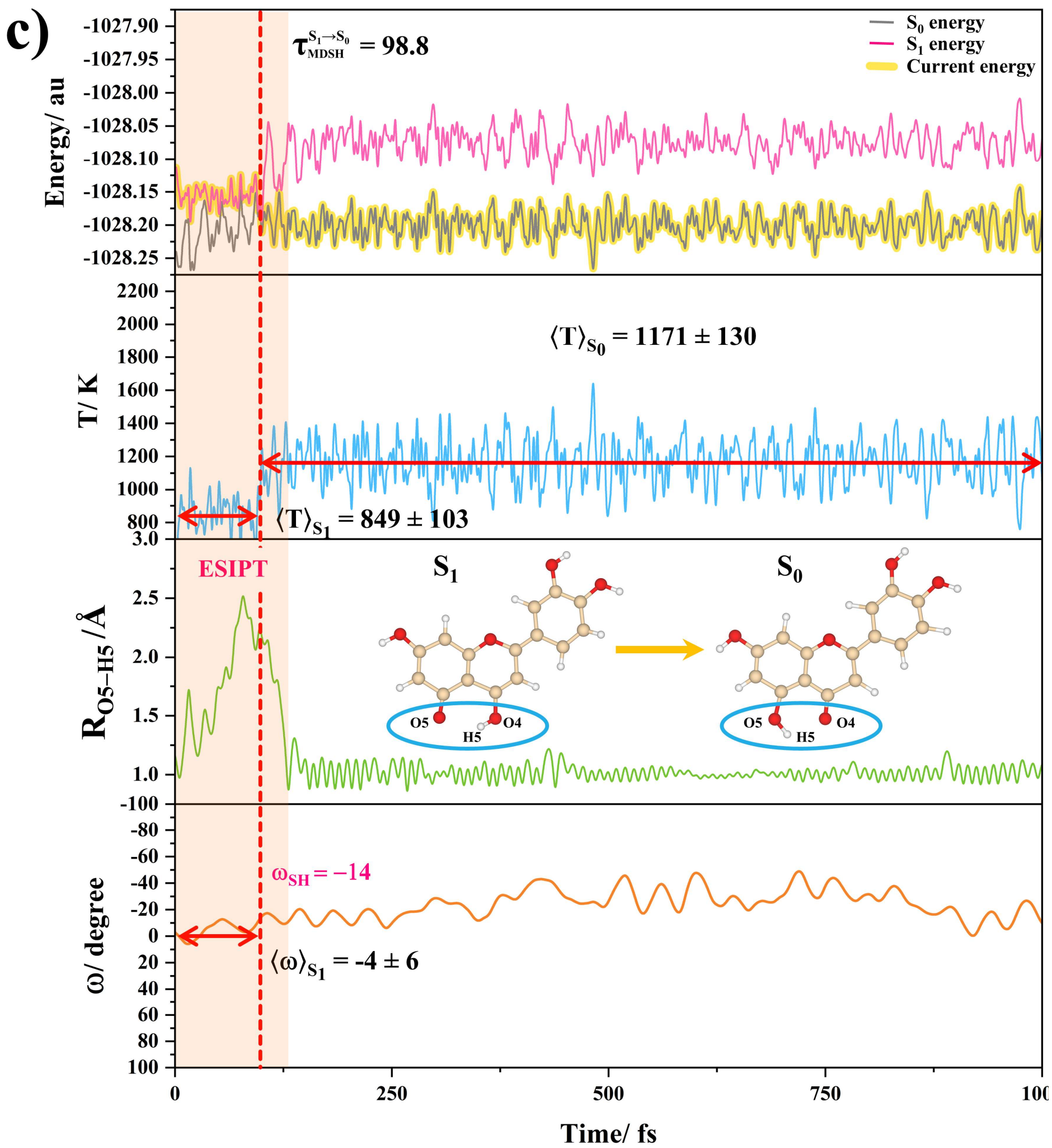

Figure S3 (Cont.)

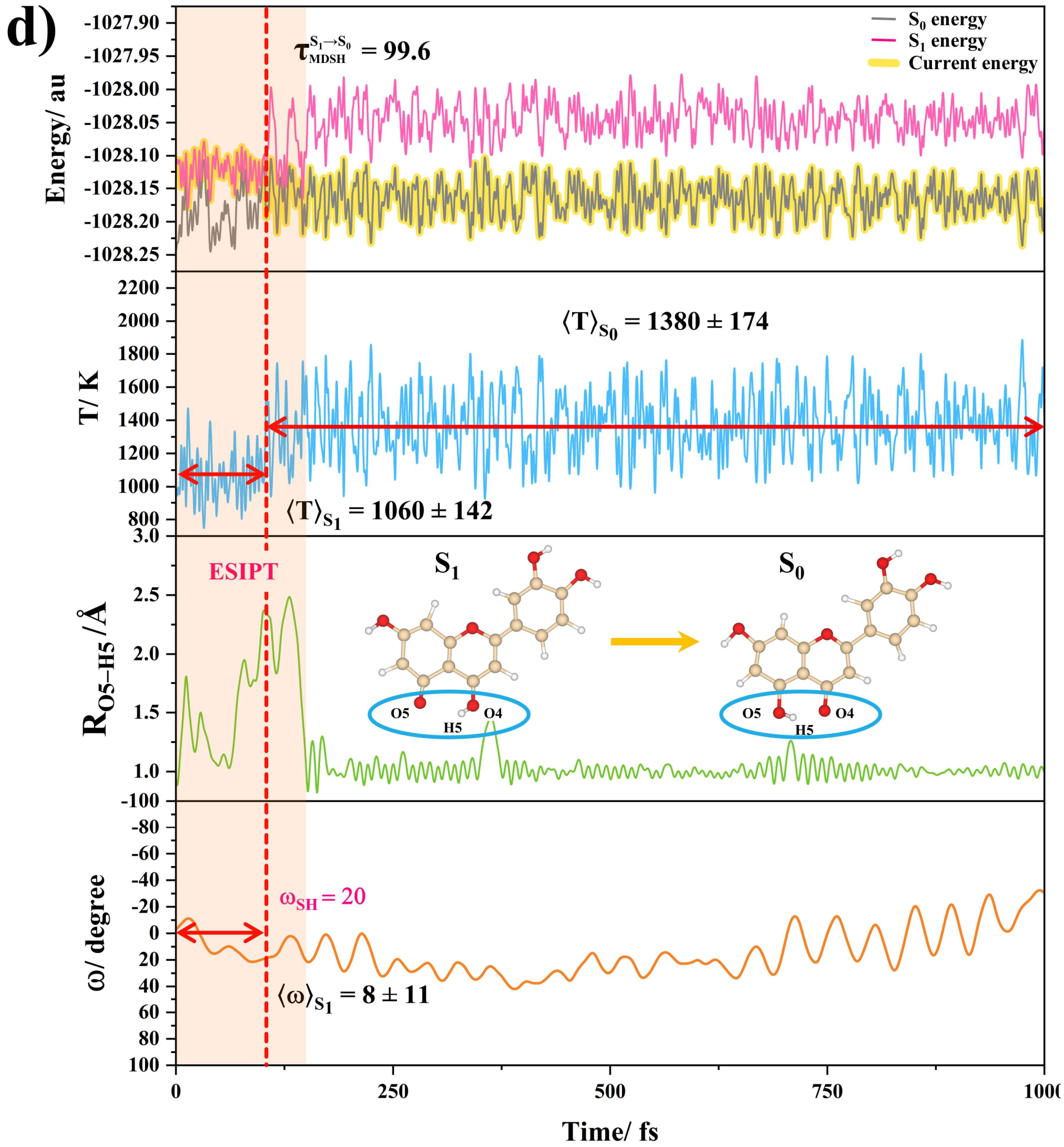

Figure S4 Vibrational spectra of Luteolin in the S_0 and S_1 states in ε = 80 and 1, highlighting the O–H stretching frequencies of the deprotonation sites.

a)–b) structure Gr–$[1]^{eq}$ and Gr–$[1]^{eq,\varepsilon}$ in the S_0 state in ε = 80 and 1, respectively.

c)–d) structure E–$[1]^{eq,*}$ and E–$[1]^{eq,*,\varepsilon}$ in the S_1 state in ε = 80 and 1, respectively.

a)

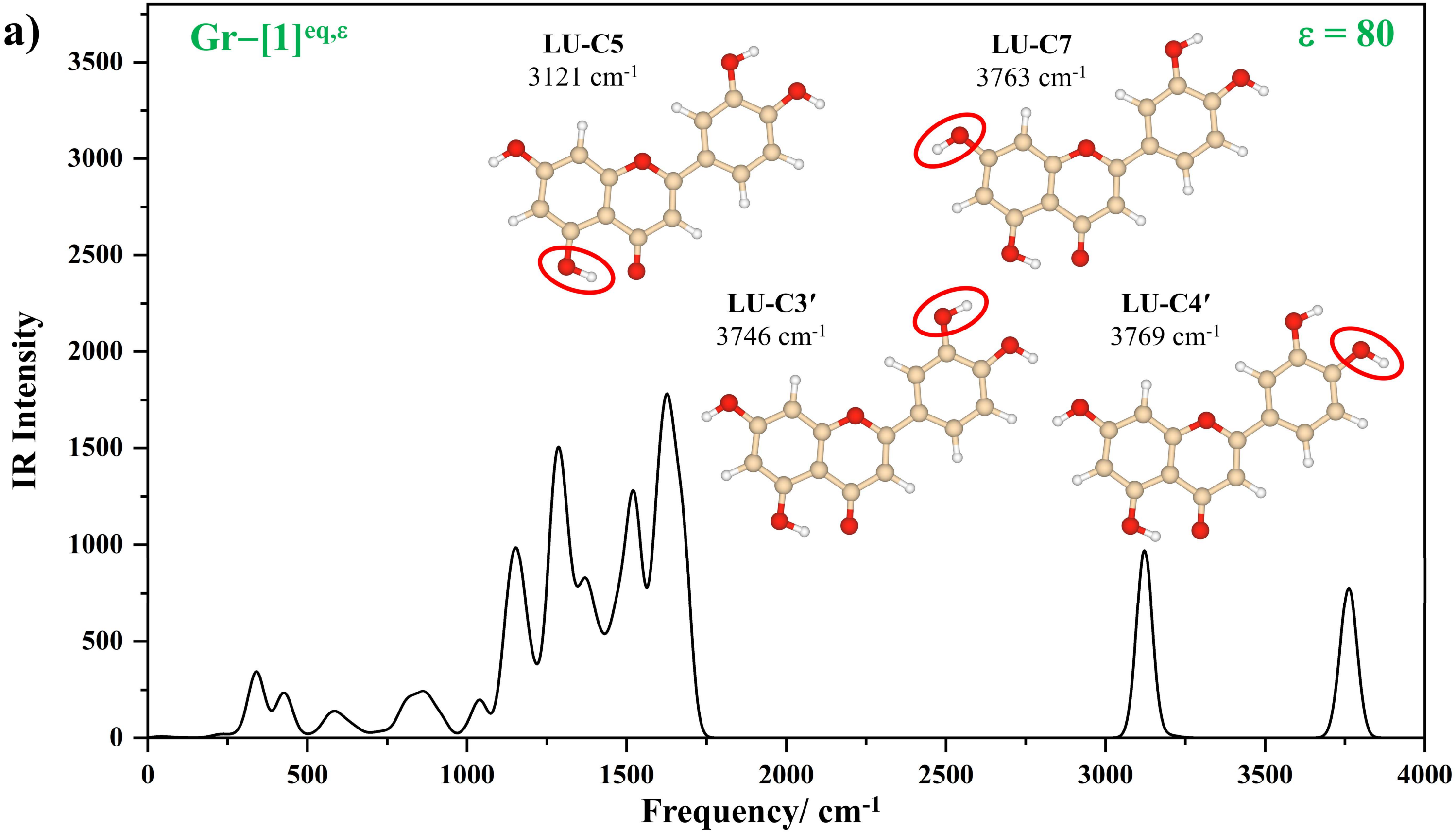

Figure S4 (Cont.)

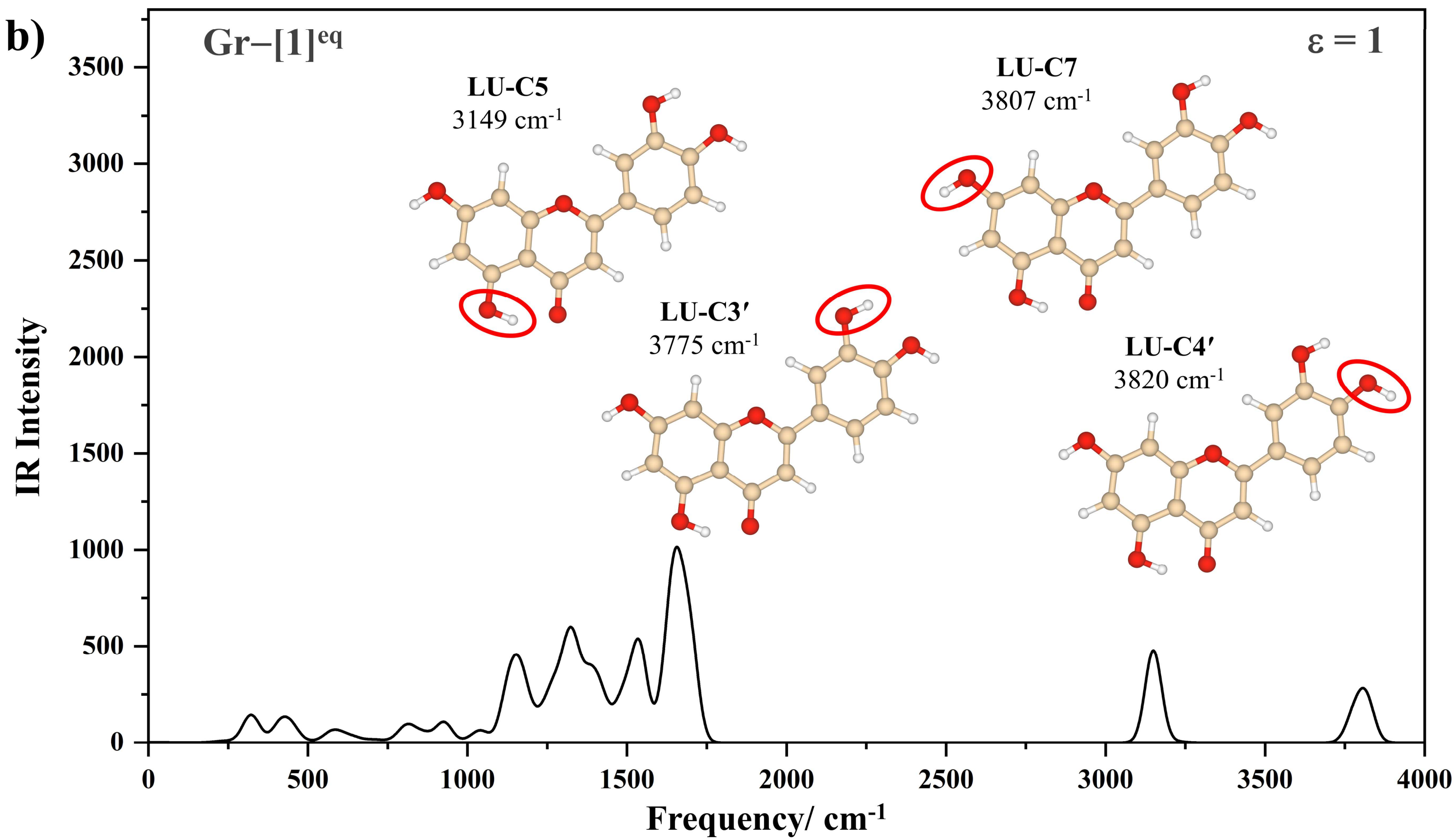

Figure S4 (Cont.)

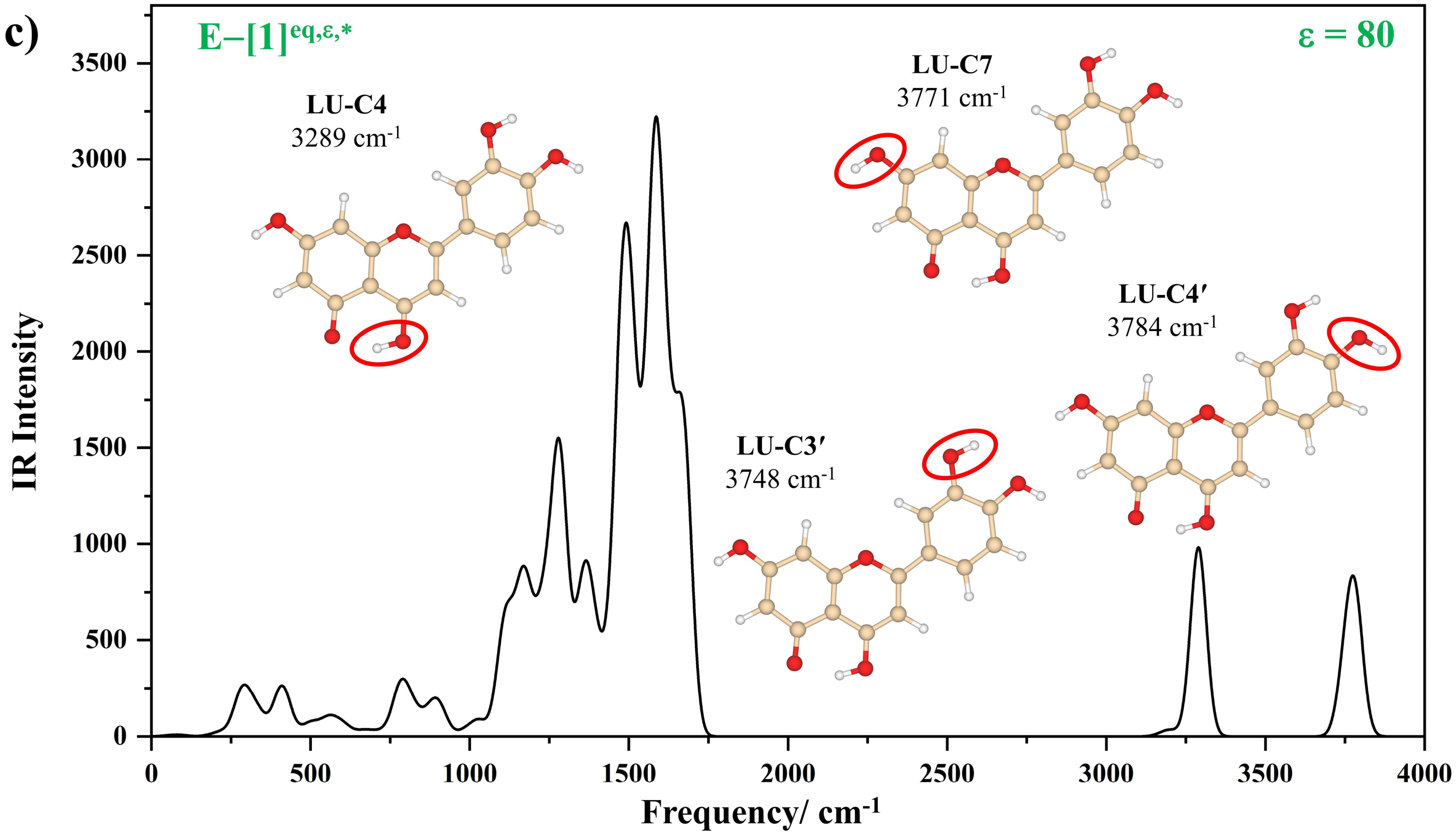

Figure S4 (Cont.)

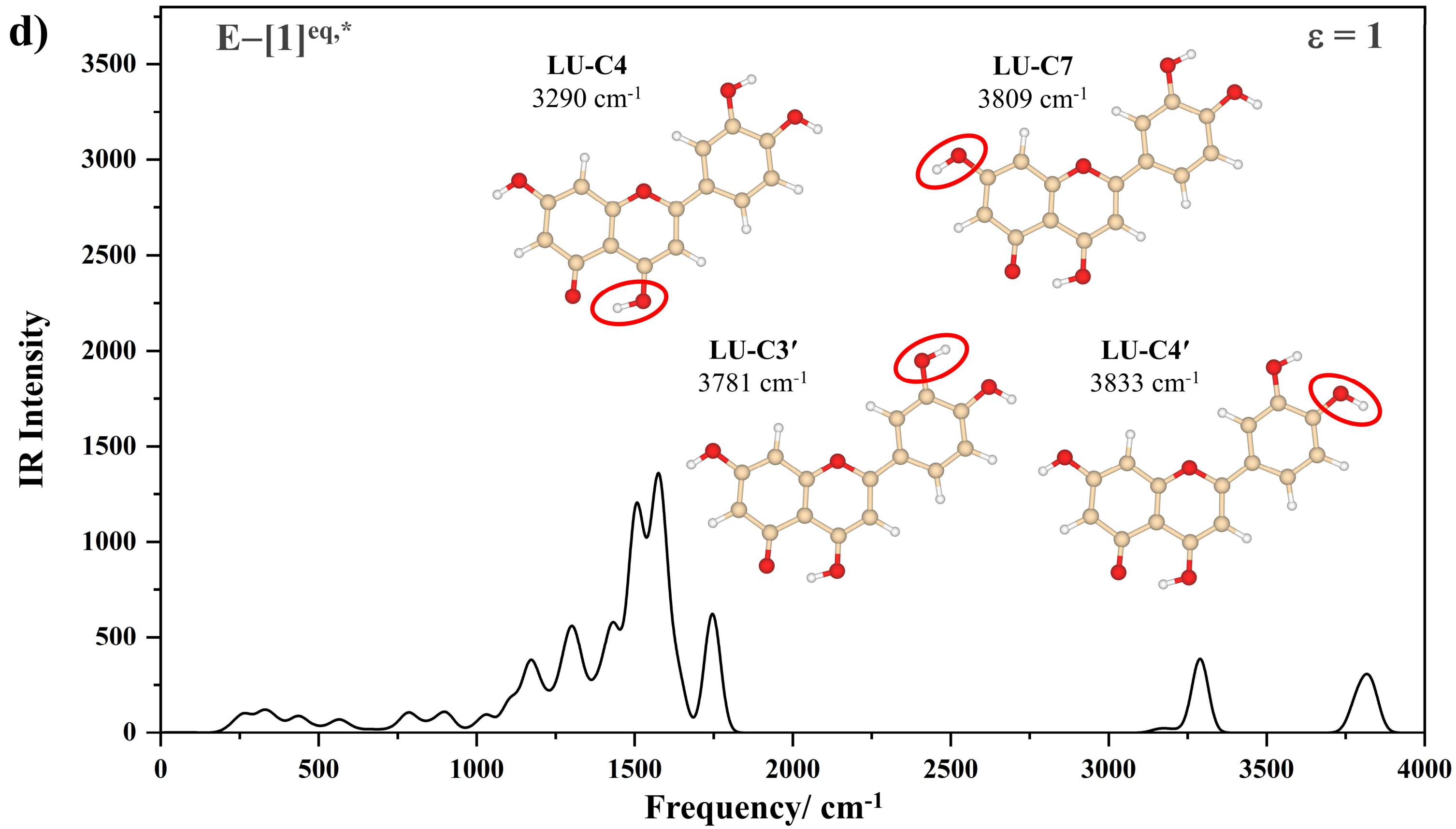

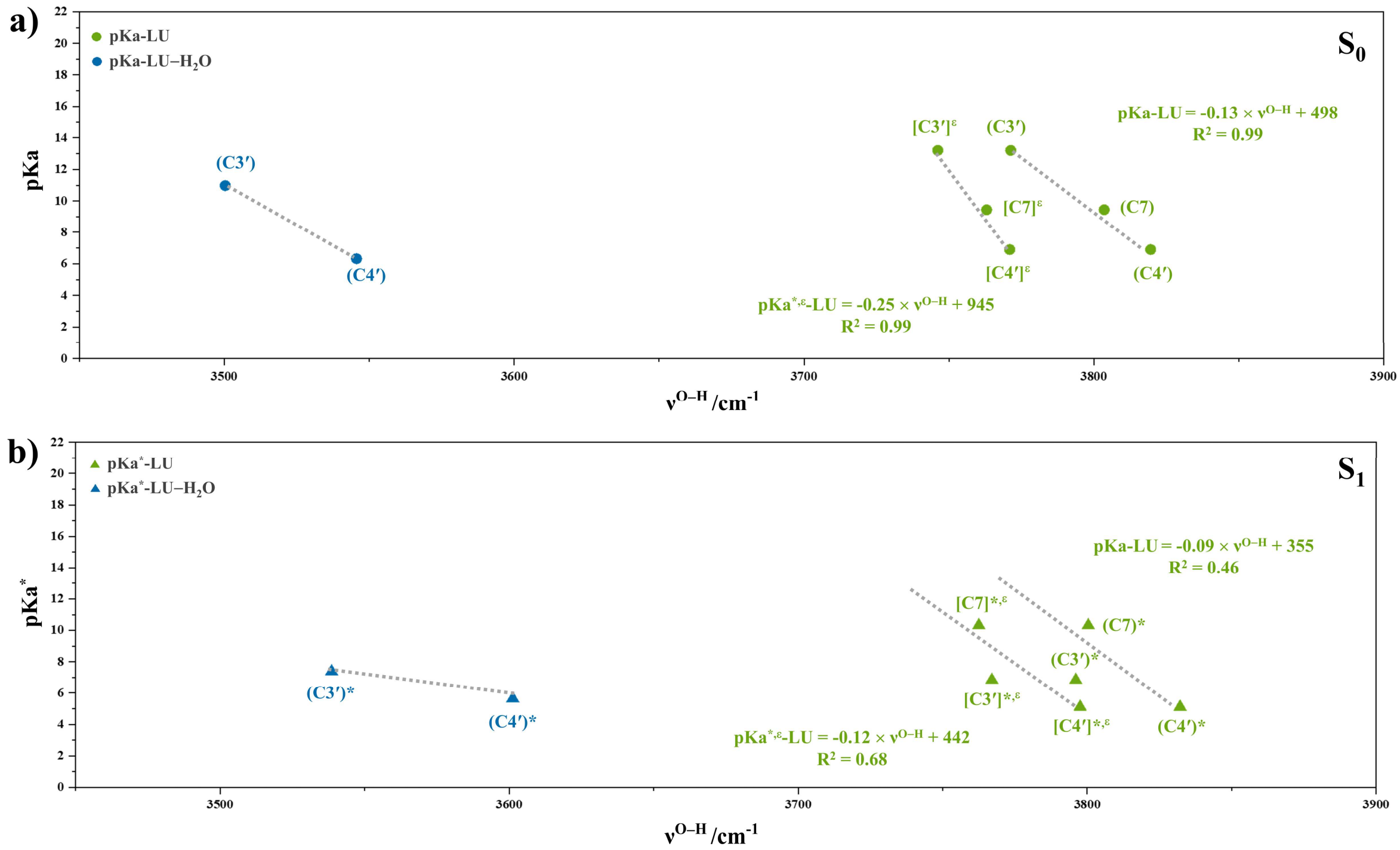

Figure S5 Separate correlations between the acidity constants obtained from the Born–Haber cycle model and the O–H stretching frequencies ($\nu^{O\text{–}H}$) at the deprotonation sites of luteolin (LU). a)–b) Correlations for the S_0-state pKa and S_1-state pKa*, respectively, computed using the isolated LU, implicit and explicit water models. (…) = isolated LU with ε = 1; [...]$^{\varepsilon}$ = implicit water model with ε = 80; * = value obtained in the S_1 state.

Table S1 Definitions of the notations and symbols used throughout the manuscript.

	Computational methods
DFT	Density functional theory
TD-DFT	Time-dependent density functional theory
CPCM	Conductor-like polarizable continuum model
NEB	Nudged elastic band
	Born–Haber cycle model
$G°_{gas,H^+}$	Free energy associated with the proton in the gas phase
$\Delta G°_{solv,H^+}$	Free energy associated with the proton in the aqueous solution
$G°_{(gas,AH)}$	Free energy of AH in the gas phase
$G°_{(gas,A^-)}$	Free energy of A^- in the gas phase
$G°_{(aq,AH)}$	Free energy of AH in aqueous solution
$G°_{(aq,A^-)}$	Free energy of A^- in aqueous solution
$\Delta G°_{(solv,AH)}$	$G°_{(aq,AH)} - G°_{(gas,AH)}$
$\Delta G°_{(gas,AH)}$	$G°_{(gas,A^-)} + G°_{(gas,H^+)} - G°_{(gas,AH)}$
$\Delta G°_{(solv,A^-)}$	$G°_{(aq,A^-)} - G°_{(gas,A^-)}$
$\Delta\Delta G°_{(solv,AH)}$	$\Delta G°_{(solv,A^-)} + \Delta G°_{(solv,H^+)} - \Delta G°_{(solv,AH)}$
$\Delta G°_{(aq,AH)}$	$\Delta G°_{(gas,AH)} + \Delta\Delta G°_{(solv,AH)}$
pKa	Ground-state acidity constant
	Förster cycle model
ΔG_{diss}	Free energy difference for proton dissociation in the S_0 state
ΔG^*_{diss}	Free energy difference for proton dissociation in the S_1 state
ΔpKa^*	Change in acidity upon $S_0 \rightarrow S_1$ excitation
pKa^*	Excited-state acidity constant
	Potential energy surface (PES)
§	Structures located at S_1/T_1 intersection
ǂ	Transition structure
ε	Structure calculated at ε = 80
[*n*]	Different conformational structure of a given LU species, distinguished primarily by the catechol-ring dihedral angle
$Gr\text{–}[n]^{eq}$	Ground state equilibrium structure
$E\text{–}[n]^{eq,*}$	Excited state equilibrium structures along the S_1 PESs
$\Delta E^{ǂ}$	Energy barrier

Table S1 (Cont.)

	Photophysical properties
$\Delta E^{S_0 \to S_1}$	$S_0 \to S_1$ vertical excitation energy
$\lambda^{S_0 \to S_1}$	$S_0 \to S_1$ excitation wavelength
$\Delta E^{S_1 \to S_0}$	$S_1 \to S_0$ relaxation energy
λ^{fluor}	Fluorescence wavelength
k^{fluor}	Fluorescence rate constant
$\Delta E^{S_1/T_1}$	Energy gap at the S_1/T_1 intersection
k^{ISC}	Intersystem crossing rate constant
$\Delta E^{T_1 \to S_0}$	$T_1 \to S_0$ relaxation energy
λ^{phos}	Phosphorescence wavelength
k^{phos}	Phosphorescence rate constant
	NVE-MDSH simulations
$\tau_{MDSH}^{S_1 \to S_0}$	$S_1 \to S_0$ surface hopping time
$\langle T \rangle_{S_1}$	Average temperature in the S_1 state
$\langle T \rangle_{S_0}$	Average temperature in the S_0 state
$\langle \omega \rangle_{S_1}$	Average dihedral angle in the S_1 state
ω_{SH}	Dihedral angle of the $S_1 \to S_0$ hopping structure

Table S2 The equilibrium structures and energies of neutral LU obtained from the DFT and TD-DFT/B3LYP/aug-cc-pVDZ methods in ε = 1 and 80, respectively.

**a) Structure**	**Energy**	**Rates of luminescence**
ω = 0.4 [-0.3] 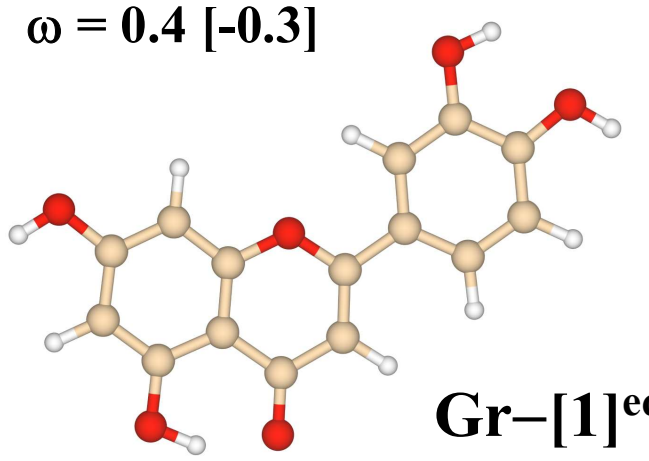 **Gr–[1]^{eq}**	E^{Tot} = -1028.5725303 [-1028.6005334]<br>$G°_{(gas,AH)}$ = -1028.3892616 [-1028.4182241]<br>$\Delta E^{S_0\rightarrow S_1}$ = 3.52 [3.55]<br>λ^{abs} = 352 [349]	-
ω = 89.8 [88.5] 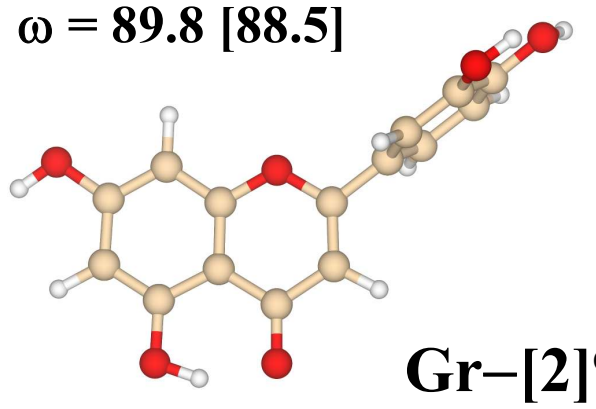 **Gr–[2]^{eq}**	E^{Tot} = -1028.5650773 [-1028.5936017]<br>$\Delta E^{S_0\rightarrow S_1}$ = 3.96 [3.76]<br>λ^{abs} = 313 [330]	-
ω = 179.5 [178.9] 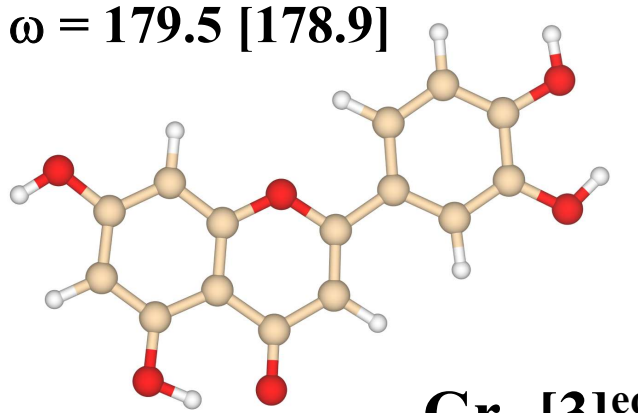 **Gr–[3]^{eq}**	E^{Tot} = -1028.5725173 [-1028.6005092]<br>$\Delta E^{S_0\rightarrow S_1}$ = 3.55 [3.59]<br>λ^{abs} = 349 [345]	-

E^{Tot} and G° = total and standard Gibbs free energies in au; $\Delta E^{S_0\rightarrow S_1}$ and $\Delta E^{S_1\rightarrow S_0}$ = vertical absorption and fluorescence energies in eV; $\Delta E^{S_1/T_1}$ = energy gap between S_1 and T_1 states in eV; $\Delta E^{T_1\rightarrow S_0}$ = phosphorescence energy in eV; k^{fluor} and k^{phos} = fluorescence and phosphorescence rates in s^{-1}, respectively; k^{ISC} = intersystem crossing rate in s^{-1}; […] = values obtained in ε = 80.

Table S2 (Cont.)

Structure	Energy	Rates of luminescence
ω = -0.1 [-1.0]<br>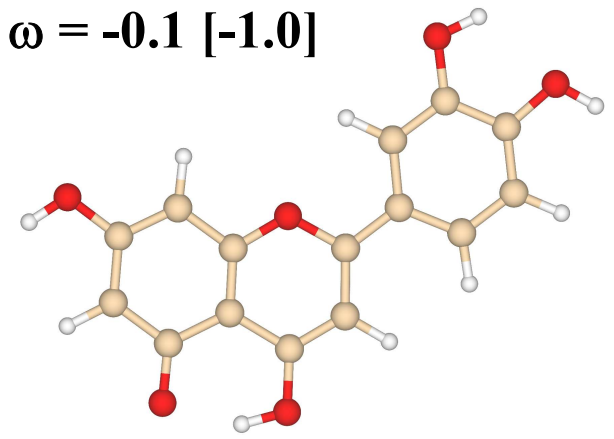<br>**E–[1]^{eq,*}**	E^{Tot} = -1028.452471 [-1028.502343]<br>$G°_{(gas,AH)}$ = -1028.4182241 [-1028.323031]<br>$\Delta E^{S_1 \rightarrow S_0}$ = -1.39 [-2.00]<br>λ^{flour} = 892 [620]<br>$\Delta E^{S_1/T_1}$ = -0.29 [-0.25]<br>$\Delta E^{T_1 \rightarrow S_0}$ = -1.11 [-1.65]<br>λ^{phos} = 1117 [751]	k^{flour} = 9.72×10^{5} [2.04×10^{7}]<br>k^{ISC} = 1.84×10^{6} [2.87×10^{5}]<br>k^{phos} = 5.32×10^{-1} [1.63×10^{0}]
ω = -178.4 [-179.8]<br>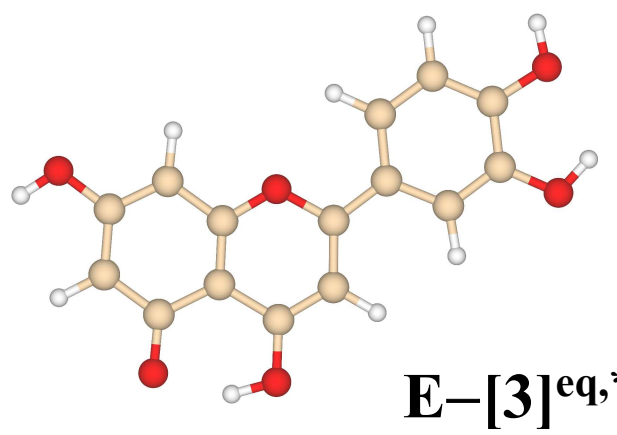<br>**E–[3]^{eq,*}**	E^{Tot} = -1028.4848292 [-1028.5019052]<br>$\Delta E^{S_1 \rightarrow S_0}$ = -1.41 [-2.00]<br>λ^{flour} = 882 [620]	-
ω = 0.7 [0.2]<br>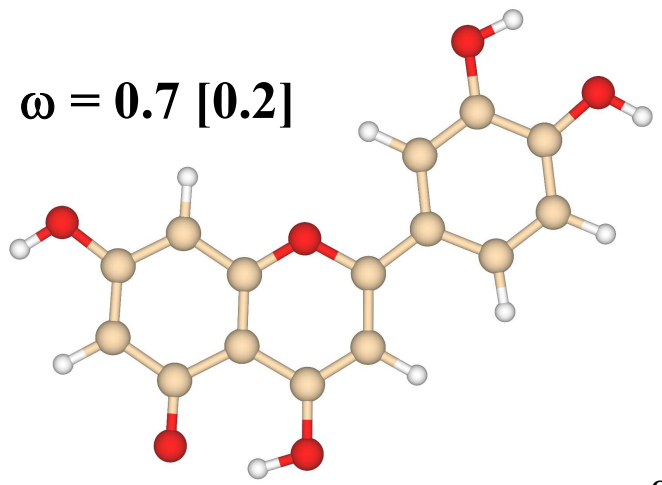<br>**E–[1]§,***	E^{Tot} = -1028.4975827 [-1028.5154396]<br>$\Delta E^{S_1 \rightarrow S_0}$ = -1.63 [-2.19]<br>λ^{flour} = 760 [566]<br>$\Delta E^{S_1/T_1}$ = -0.39 [-0.41]<br>$\Delta E^{T_1 \rightarrow S_0}$ = -1.24 [-1.78]<br>λ^{phos} = 1000 [696]	k^{flour} = 2.25×10^{6} [3.18×10^{7}]<br>k^{ISC} = 5.82×10^{7} [2.59×10^{7}]<br>k^{phos} = 6.39×10^{-1} [1.56×10^{0}]

Table S3 The equilibrium structures and standard Gibbs free energies of the deprotonated forms of LU obtained from the DFT and TD-DFT/B3LYP/aug-cc-pVDZ methods in ε = 1 and 80, respectively. $G^{\circ}_{(gas,A^{-})}$ = standard Gibbs free energy of the deprotonated LU in au; […] = value obtained in ε = 80.

	LU	LU*
**LU-C4'**	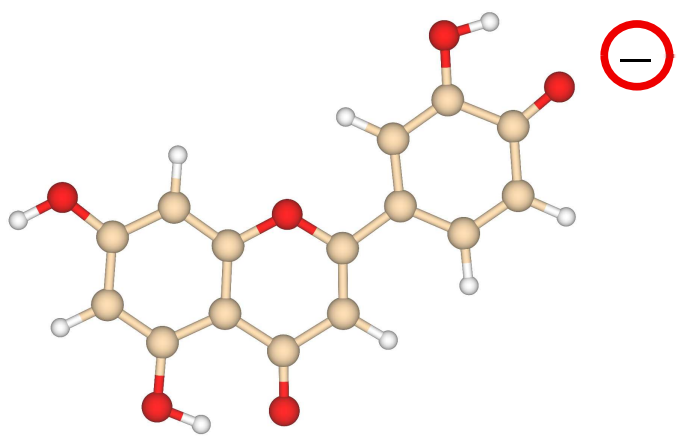 **$G^{\circ}_{(gas,A^{-})}$** = -1027.8880366 [-1027.9687587]	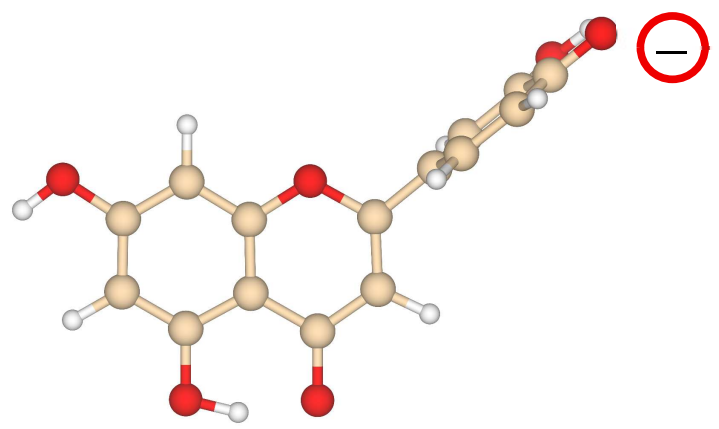 **$G^{\circ}_{(aq,A^{-})}$** = -1027.7990637 [-1027.8772869]
**LU-C3'**	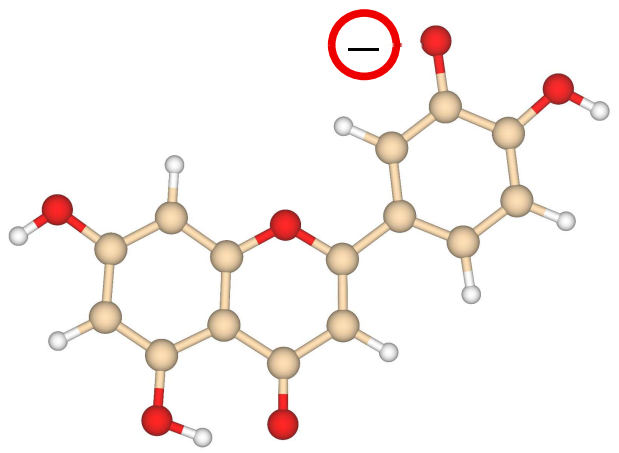 **$G^{\circ}_{(gas,A^{-})}$** = -1027.8565916 [-1027.955129]	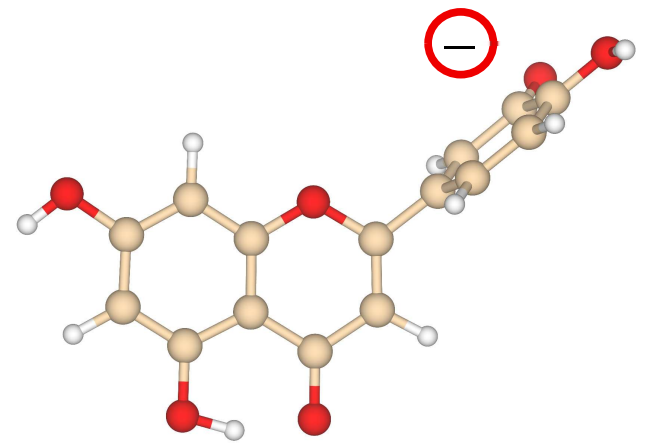 **$G^{\circ}_{(gas,A^{-})}$** = -1027.811586 [-1027.8727051]
**LU-C7**	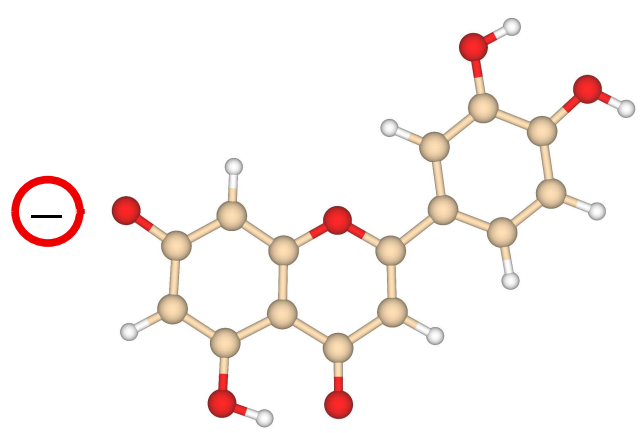 **$G^{\circ}_{(gas,A^{-})}$** = -1027.8648728 [-1027.9632311]	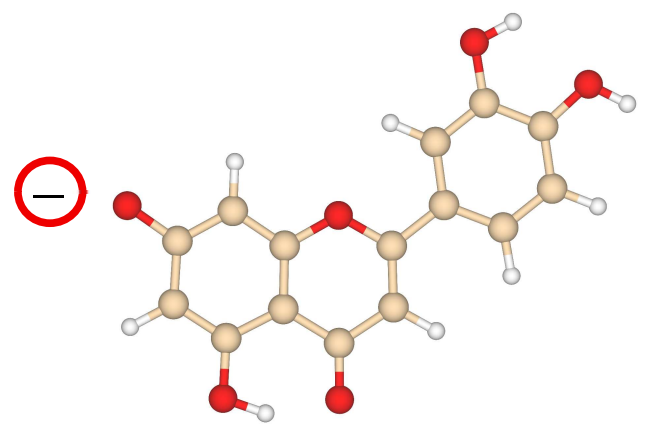 **$G^{\circ}_{(gas,A^{-})}$** = -1027.7982149 [-1027.870405]
**LU-C5**	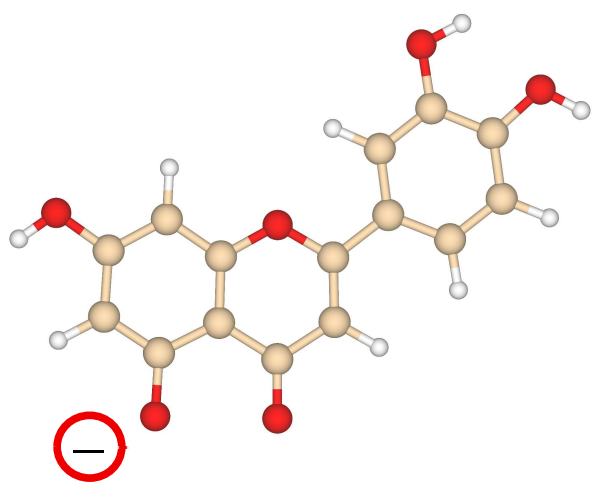 **$G^{\circ}_{(gas,A^{-})}$** = -1027.8374511 [-1027.9487400]	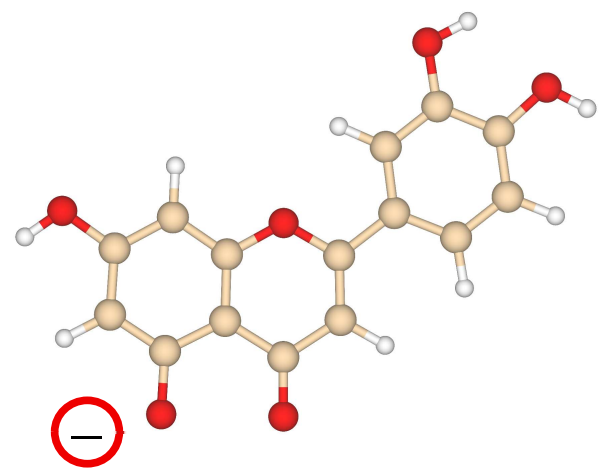 **$G^{\circ}_{(gas,A^{-})}$** = -1027.7735271 [-1027.8469745]

Table S4 Natural transition orbital (NTO) analysis of the low-lying singlet excited states of LU for representative structures along the S_1 PES. a) Structures Gr–$[1]^{eq}$ and Gr–$[2]^{eq}$ at ε = 1. b) Structures Gr–$[1]^{eq,ε}$ and Gr–$[2]^{eq,ε}$ at ε = 80. c) Structures E–$[1]^{eq,*}$ and E–$[1]^{eq,ε,*}$ (ω ≈ 0°) following O5–H5→O4 ESIPT at ε = 1 and 80, respectively. The dominant orbital transitions and their percentage contributions are included. ΔE^{ex} = vertical excitation energy in eV; λ^{abs} = absorption wavelength in nm; (…) = percentage contributions.

a)	State	λ^{abs}	ΔE^{ex}	Major Orbital Contributions			
**Gr–$[1]^{eq}$ (ω = 0°)**	S1	352	3.52	73→74(98.1)	72→75(1.2)		
	S2	316	3.92	73→74(99.9)			
	S3	311	3.99	73→74(95.7)	72→75(2.8)		
	S4	298	4.16	73→74(82.9)	72→75(14.3)	71→76(1.9)	
	S5	276	4.49	73→74(72.6)	72→75(21.9)	71→76(4.4)	
	S6	266	4.66	73→74(99.8)			
	S7	264	4.69	73→74(75.4)	72→75(20.7)	71→76(2.8)	
	S8	251	4.94	73→74(68.4)	72→75(21.9)	71→76(8.4)	
	S9	250	4.96	73→74(92.8)	72→75(4.3)	71→76(1.5)	
	S10	243	5.11	73→74(89.0)	72→75(10.8)		
**Gr–$[2]^{eq}$ (ω = 90°)**	S1	313	3.97	73→74(96.5)	72→75(2.6)		
	S2	304	4.07	73→74(99.0)			
	S3	293	4.23	73→74(98.8)	72→75(1.1)		
	S4	275	4.51	73→74(61.5)	72→75(37.5)		
	S5	257	4.83	73→74(82.3)	72→75(11.4)	71→76(4.1)	70→77(1.8)
	S6	256	4.85	73→74(74.4)	72→75(18.4)	71→76(5.7)	70→77(1.3)
	S7	254	4.88	73→74(97.9)	72→75(1.5)		
	S8	252	4.92	73→74(80.5)	72→75(12.5)	71→76(5.3)	70→77(1.4)
	S9	242	5.11	73→74(76.8)	72→75(15.9)	71→76(6.0)	
	S10	241	5.14	73→74(67.0)	72→75(28.9)	71→76(3.0)	

Table S4 (Cont.)

b)	State	λ^{abs}	ΔE^{ex}	Major Orbital Contributions			
	S1	367	3.38	73→74(98.4)			
	S2	332	3.73	73→74(98.1)			
	S3	304	4.08	73→74(92.0)	72→75(5.8)	71→76(1.3)	
	S4	299	4.14	73→74(99.9)			
**Gr−[1]$^{eq,\varepsilon}$ (ω = 0°)**	S5	288	4.30	73→74(84.2)	72→75(11.9)	71→76(2.6)	
	S6	272	4.56	73→74(87.7)	72→75(10.2)		
	S7	261	4.75	73→74(88.4)	72→75(8.4)	71→76(2.3)	
	S8	252	4.93	73→74(63.8)	72→75(24.6)	71→76(9.8)	
	S9	247	5.03	73→74(98.7)	72→75(1.0)		
	S10	240	5.16	73→74(48.1)	72→75(27.6)	71→76(21.5)	70→77(1.8)
	S1	330	3.76	73→74(99.7)			
	S2	319	3.88	73→74(97.5)	72→75(1.5)		
	S3	289	4.30	73→74(99.6)			
	S4	280	4.43	73→74(80.6)	72→75(18.2)		
**Gr−[2]$^{eq,\varepsilon}$ (ω = 90°)**	S5	274	4.52	73→74(99.4)			
	S6	261	4.75	73→74(91.2)	72→75(7.1)	71→76(1.2)	
	S7	252	4.92	73→74(74.0)	72→75(21.7)	71→76(1.4)	70→77(1.2)
	S8	251	4.95	73→74(85.9)	72→75(11.3)	71→76(1.8)	
	S9	242	5.13	73→74(99.5)			
	S10	238	5.22	73→74(97.8)	72→75(1.3)		

Table S4 (Cont.)

c)	State	λ^{abs}	ΔE^{ex}	Major Orbital Contributions		
**E−[1]^{eq,*} (ω = 0°)**	S1	890	1.39	73→74(99.5)		
	S2	471	2.63	73→74(100.0)		
	S3	407	3.05	73→74(88.4)	72→75(10.7)	
	S4	372	3.33	73→74(90.9)	72→75(8.7)	
	S5	354	3.50	73→74(77.7)	72→75(20.6)	
	S6	347	3.58	73→74(100.0)		
	S7	335	3.71	73→74(77.0)	72→75(17.1)	71→76(4.8)
	S8	301	4.11	73→74(99.8)		
	S9	300	4.13	73→74(86.6)	72→75(8.7)	71→76(3.1)
	S10	291	4.27	73→74(99.8)		
**E−[1]$^{eq,\varepsilon,*}$ (ω = 0°)**	S1	621	2.00	73→74(99.4)		
	S2	410	3.02	73→74(98.0)		
	S3	368	3.37	73→74(100.0)		
	S4	352	3.52	73→74(93.0),	72→75(5.4)	
	S5	332	3.74	73→74(67.2),	72→75(31.0)	71→76(1.0)
	S6	310	4.00	73→74(68.6),	72→75(27.5)	71→76(2.6)
	S7	286	4.33	73→74(97.0),	72→75(2.6)	
	S8	276	4.49	73→74(99.6)		
	S9	262	4.73	73→74(68.0),	72→75(22.8)	71→76(8.0)
	S10	258	4.81	73→74(99.0)		

Calculations on kinetics and thermodynamics of reaction pathways

The kinetics and thermodynamics of the photoluminescence pathway were investigated by calculating the quantized-vibrational rate constants ($k^{Q\text{-}vib}$) over the temperature range of 270–360 K. The $k^{Q\text{-}vib}$ values were evaluated using Eq. (1),[1] where $\Delta E^{‡,ZPC}$ represents the zero-point-energy-corrected activation barrier. This quantity was obtained by applying the zero-point energy correction (ΔE^{ZPE}) to the corresponding energy barrier ($\Delta E^{‡}$) determined from the NEB calculations.

$$k^{Q\text{-}vib}(T) = \frac{k_B T}{\hbar} \frac{Q^{‡,ZPC}}{Q^{R,ZPC}} e^{-\Delta E^{‡,ZPC}/k_B T} \quad (1)$$

where $Q^{R,ZPC}$ and $Q^{‡,ZPC}$ are the partition functions of the precursor and transition structures, respectively, and k_B and $\hbar$ are the Boltzmann and Planck constants, respectively.

The activation free energies ($\Delta G^{‡}$) were subsequently derived from $k^{Q\text{-}vib}$ using the corresponding transition-state-theory relationship. The activation enthalpies ($\Delta H^{‡}$) were determined using Eq. (2),

$$\ln k^{Q\text{-}vib}(T) = \ln A + \frac{\Delta S^{‡}}{R} - \frac{\Delta H^{‡}}{RT}. \quad (2)$$

where $\Delta S^{‡}$ is the activation entropy and R is the gas constant. The $\Delta H^{‡}$ values were obtained from the linear relationship between $\ln k^{Q\text{-}vib}$ and 1000/T.

Because calculations of the TD-DFT Hessian matrix of LU using the aug-cc-pVDZ basis set were computationally demanding, the Hessian calculations were instead performed using the def2-SVP basis set implemented in ORCA. The def2-SVP basis set is a polarized split-valence double-ζ basis set and was selected to provide a practical balance between computational accuracy and cost for these calculations. The energy barriers ($\Delta E^{‡}$) obtained based on the TD-DFT/B3LYP/ def2-SVP method are slightly higher than those obtained from the TD-DFT/B3LYP/aug-cc-pVDZ method.

The kinetic and thermodynamic quantities were evaluated using the DL-FIND program[2] implemented within the ChemShell software package.[3] Based on the photoluminescence pathways presented in Figure 5, the kinetics and thermodynamics of the individual reaction pathways were examined. For example, for the $E\text{–}[1]^{§,\varepsilon,*} \rightarrow E\text{–}[2]^{‡,\varepsilon,*} \rightarrow E\text{–}[3]^{eq,\varepsilon,*}$ pathway shown in Figure 5a, the corresponding activation free energies ($\Delta G^{‡}$), activation enthalpies ($\Delta H^{‡}$), and rate constants ($k^{Q\text{-}vib}$) were obtained using the TST treatment and are summarized in Table S5†.

1. D. S. Sholl and J. A. Steckel, *Density Functional Theory: A Practical Introduction*, John Wiley & Sons, 2009.
2. J. Kästner, J. M. Carr, T. W. Keal, W. Thiel, A. Wander and P. Sherwood, *J. Phys. Chem. A,* 2009**, 113**, 11856–11865.
3. ChemShell, a Computational Chemistry Shell, www.chemshell.org, (accessed August 2026)

Table S5 a)–b) Thermodynamic and kinetic properties on the S_1 PES for the large-amplitude rotation of ω from ~0 to ~180° with ε = 80 and ε = 1, respectively. Rate constants, temperatures and energies are in s^{-1}, K and kJ/mol, respectively. $\Delta E^{‡}$ = energy barrier on the S_1 PES obtained from the TD-DFT/B3LYP/def2-SVP method; $\Delta E^{‡,ZPC}$ = zero-point energy-corrected $\Delta E^{‡}$, obtained by including the zero-point correction energy to $\Delta E^{‡}$; $\Delta H^{‡}$, $\Delta S^{‡}$ and $\Delta G^{‡}$ = activation enthalpy, entropy and Gibbs free energies, respectively; T_c = crossover temperature; T = temperature; $k^{Q\text{-}vib}$= rate constant obtained with quantized vibrations including the zero-point vibrational energy.

	$\Delta E^{‡}$	ΔE^{ZPE}	$\Delta E^{‡,ZPC}$	$\Delta H^{‡}$	T_c	T	$k^{Q\text{-}vib}$	$\Delta G^{‡}$	$\Delta S^{‡}$
**E–[1]^{§,ε,*}→E–[2]^{‡,ε,*}→E–[3]^{§,ε,*}**	43.5	-2.0	41.5	42.5	79	270	8.79×10^{4}	44.9	-8.82×10^{-3}
						278	1.15×10^{4}	45.0	-9.32×10^{-3}
						286	2.58×10^{4}	45.2	-9.86×10^{-3}
						295	4.42×10^{4}	45.3	-1.04×10^{-2}
						304	7.58×10^{4}	45.5	-1.10×10^{-2}
						314	1.30×10^{5}	45.7	-1.17×10^{-2}
						324	2.22×10^{5}	45.9	-1.24×10^{-2}
						360	1.12×10^{6}	46.6	-1.49×10^{-2}

	$\Delta E^{‡}$	ΔE^{ZPE}	$\Delta E^{‡,ZPC}$	$\Delta H^{‡}$	T_c	T	$k^{Q\text{-}vib}$	$\Delta G^{‡}$	$\Delta S^{‡}$
**E–[1]§,*→E–[2]‡,*→E–[3]§,***	52.8	-6.0	46.8	48.9	83	270	2.18×10^{3}	45.9	1.11×10^{-2}
						278	4.18×10^{4}	45.9	1.11×10^{-2}
						286	8.04×10^{4}	45.9	1.12×10^{-2}
						295	1.54×10^{4}	45.8	1.13×10^{-2}
						304	2.97×10^{4}	45.8	1.13×10^{-2}
						314	5.70×10^{5}	45.8	1.14×10^{-2}
						324	1.09×10^{5}	45.8	1.14×10^{-2}
						360	7.75×10^{6}	45.7	1.16×10^{-2}

Table S6 The O–H stretching frequencies ($\nu^{O\text{–}H}$) of the deprotonation sites in the S_0 and S_1 states, obtained based on the three computational models.
a) Isolated LU (ε = 1) and implicit water models (ε = 80).
b) Explicit water model (1:1 LU–H_2O complexes). (A) and (B) = structure with and without the O5–H5→O4 intramolecular H-bond and ESIPT, respectively.

**a)**	**LU (A)**			
	S_0		S_1	
	**ε = 1**	**ε = 80**	**ε = 1**	**ε = 80**
**LU-C5**	3147	3128	3300	3290
**LU-C3'**	3771	3746	3796	3767
**LU-C7**	3804	3763	3800	3763
**LU-C4'**	3820	3771	3832	3798

**b)**	**LU–H_2O (A)**		**LU–H_2O (B)**	
	S_0	S_1	S_0	S_1
**LU-C5**	3217	3200	3581	3560
**LU-C3'**	3500	3539	3510	3551
**LU-C7**	3577	3560	3586	3570
**LU-C4'**	3546	3601	3595	3641
